# Impact of Isolation Techniques on the Content of Small Extracellular Vesicles

**DOI:** 10.1002/jev2.70290

**Published:** 2026-05-28

**Authors:** Yue Su, Kekoolani S. Visan, Sarah Voss, Marta Prieto‐Vila, Juntaro Matsuzaki, Jason Ying Kuen Chan, Judy Wai Ping Yam, Andreas Möller

**Affiliations:** ^1^ Department of Otorhinolaryngology, Faculty of Medicine The Chinese University of Hong Kong Shatin Hong Kong SAR; ^2^ JC STEM Lab of Personalised Cancer Medicine, Li Ka Shing Institute of Health Sciences The Chinese University of Hong Kong Hong Kong SAR; ^3^ Faculty of Health, School of Biomedical Sciences Queensland University of Technology Brisbane Queensland Australia; ^4^ QIMR Berghofer Medical Research Institute Brisbane Queensland Australia; ^5^ Division of Interdisciplinary Genetics and Nanomedicine, Research Center for Drug Discovery Keio University Faculty of Pharmacy Tokyo Japan; ^6^ Human Biology‐Microbiome‐Quantum Research Center (WPI‐Bio2Q) Keio University Tokyo Japan; ^7^ Department of Pathology, School of Clinical Medicine, Li Ka Shing Faculty of Medicine The University of Hong Kong Hong Kong SAR; ^8^ State Key Laboratory of Liver Research The University of Hong Kong Hong Kong SAR; ^9^ Materials Innovation Institute for Life Sciences and Energy HKU‐SIRI Shenzhen China

## Abstract

An important topic of discussion amongst the extracellular vesicle (EV) research field is which genetic materials are considered true constituents of EV cargo. What were once regarded as non‐EV components have now evolved to be potentially essential to EV composition, serving as key mediators in communication. Researchers continue to pursue varying methods for the isolation of EVs with the desired content. However, the desired content depends on the source and the requirements of the intended downstream use of EVs. Different isolation methods can modify EV cargo, impacting functional EV‐induced effects and analysis of EV contents. Ensuring that any result produced is truly representative of EVs and not of co‐isolated materials is imperative.

Here, we describe a side‐by‐side comparison of the outcomes of EV isolation from plasma of healthy individuals, using size exclusion chromatography and an ultrafast filtration system. Characterisation of EVs was performed by particle (nanoparticle tracking analysis), protein (Bradford assay) and lipid (sulfo‐phospho‐vanillin assay) quantification, morphology visualisation (transmission electron microscopy), microRNA expression (miRNA sequencing) and assessment of protein absence or presence (traditional and capillary‐based western blot analyses, ELISA and mass spectrometry). To our surprise, the isolation methods employed identified significant differences in the protein abundance and composition of the resulting EVs. SEC isolated plasma EVs with high abundance of EV transmembrane and cytosolic markers, as well as corona‐related complement, fibrinogen and extracellular matrix proteins. However, this was accompanied by high quantities of non‐EV immunoglobulins. In contrast, UFF isolated plasma EVs with high abundance of EV integrins and corona‐related complement proteins, albeit with high lipoprotein abundance. Each isolation approach produces EVs with different biomolecular properties, which might provide specific advantages and disadvantages, depending on the intended use of EVs. Therefore, isolation methods need to be tested intensively and selected carefully for downstream applications.

## 1 Introduction

Extracellular vesicles (EVs) (Ham et al. 2025) define a broad category of nano‐ to micro‐sized, phospholipid‐bound particles that encase an assortment of biomolecular cargo, derived from their cell‐of‐origin (Doyle and MZ. 2019). The unique composition of EVs and presence in all bodily fluids enable their crucial roles in intercellular communication (Moller and Lobb 2020; Ragni 2025). The combination of their stable nature and their omnipresence indicates their potential to act as powerful biomarkers of disease, as well as tools for cell‐targeting therapies and drug delivery (Du et al. 2023). Blood plasma is the most common source of bodily fluid EVs used for medical research (Lucien et al. 2023; Williams et al. 2023), due to their versatility in clinical applications (Taha and Bogoniewski 2023; Zhao et al. 2024). However, the method/s employed for plasma EV isolation largely depends on the site‐specific protocols established and used, often resulting from opportunistic and historical approaches, depending on platform availability. Optimisation for the intended use of the EVs in downstream applications is often an afterthought. In the context of biomarker discovery, an EV isolation method that enables easy biomarker detection while preserving the biomarker target, using a stand‐alone enrichment strategy, would be ideal. For the use of EVs as drug delivery carriers and therapeutic agents, an isolation method that preserves specific surface proteins that prolong circulation and enhance uptake by target cells would be optimal. It is imperative that the EVs isolated are as ‘pure’ as possible. With the ever‐evolving definition of ‘EVs’, the term ‘purity’ is therefore fully dependent on context, hence it can be assessed by various measures (Ciferri et al. 2021; Dong et al. 2020; Holcar et al. 2020; Veerman et al. 2021).

Traditional EV nomenclature is predicated on biogenesis and size‐based classification, giving rise to different EV subtypes (Abels and Breakefield 2016; Zhang et al. 2018). However, as current EV isolation approaches cannot separate EVs based on biogenesis pathways, it is generally accepted for EVs to be classified by size (Welsh et al. 2024). Small EVs (sEVs) range from 30 to 200 nm in diameter, whereas large EVs (lEVs) are those greater than 200 nm (Pezzicoli et al. 2020). Due to a continuum and therefore overlap in size, sEV populations contain a mixture of EVs of endosomal and exocytic origin (Veerman et al. 2021; Zhang et al. 2018; Lim et al. 2021), which vary in their cargo and can therefore impact analyses.

Separation of sEVs from other EV subtypes, such as lEVs, or even non‐vesicular extracellular particles (NVEPs) is essential as NVEPs and EV subtypes differ in their capacity to elicit responses in target cells (Wahlund et al. 2017). Ineffective elimination of contaminating vesicles may result in a reduction or alteration of bioactivity (Kronstadt et al. 2023). The cargo of sEVs is derived from the plasma membrane and cytosol of the parental cell (Chen et al. 2021). Hence, detection of materials from the endoplasmic reticulum, Golgi apparatus, mitochondria and nucleus are likely indicative of contamination. This poses issues for multi‐omics analyses, as presence of other intracellular compartments may generate false positive findings (Charest 2020; Geyer et al. 2019; Shaba et al. 2022).

Currently, there are discussions surrounding the existence and composition of a biomolecular corona on EVs (Heidarzadeh et al. 2023; Tóth et al. 2021; Ragni and Taiana 2024), which are proteins, nucleic acids and potentially other material, acquired from its intracellular formation or upon exposure to the extracellular milieu, and transiently attached to the EV surface (Esmaeili et al. 2025). Proteins both tightly and loosely anchored to the outside of sEVs have been reported to impact the functionality of sEVs (Liam‐Or et al. 2024; Dietz et al. 2023; Lima et al. 2021; Wolf et al. 2022). Certain proteins that were once considered contamination, such as apolipoproteins and albumin, have now been reported to be essential elements of the corona (Esmaeili et al. 2025; Mariam et al. 2016; Francia et al. 2020). Assessing the presence of such proteins indicates the capacity of isolation methods to preserve sEV integrity (Paget et al. 2022; Visan et al. 2022). On the contrary, the presence of corona proteins may mask intravesicular or transmembrane proteins, which can impact biomarker detection as well as uptake by recipient cells (Takov et al. 2017; Nieuwland and Siljander 2024).

**FIGURE 1:**
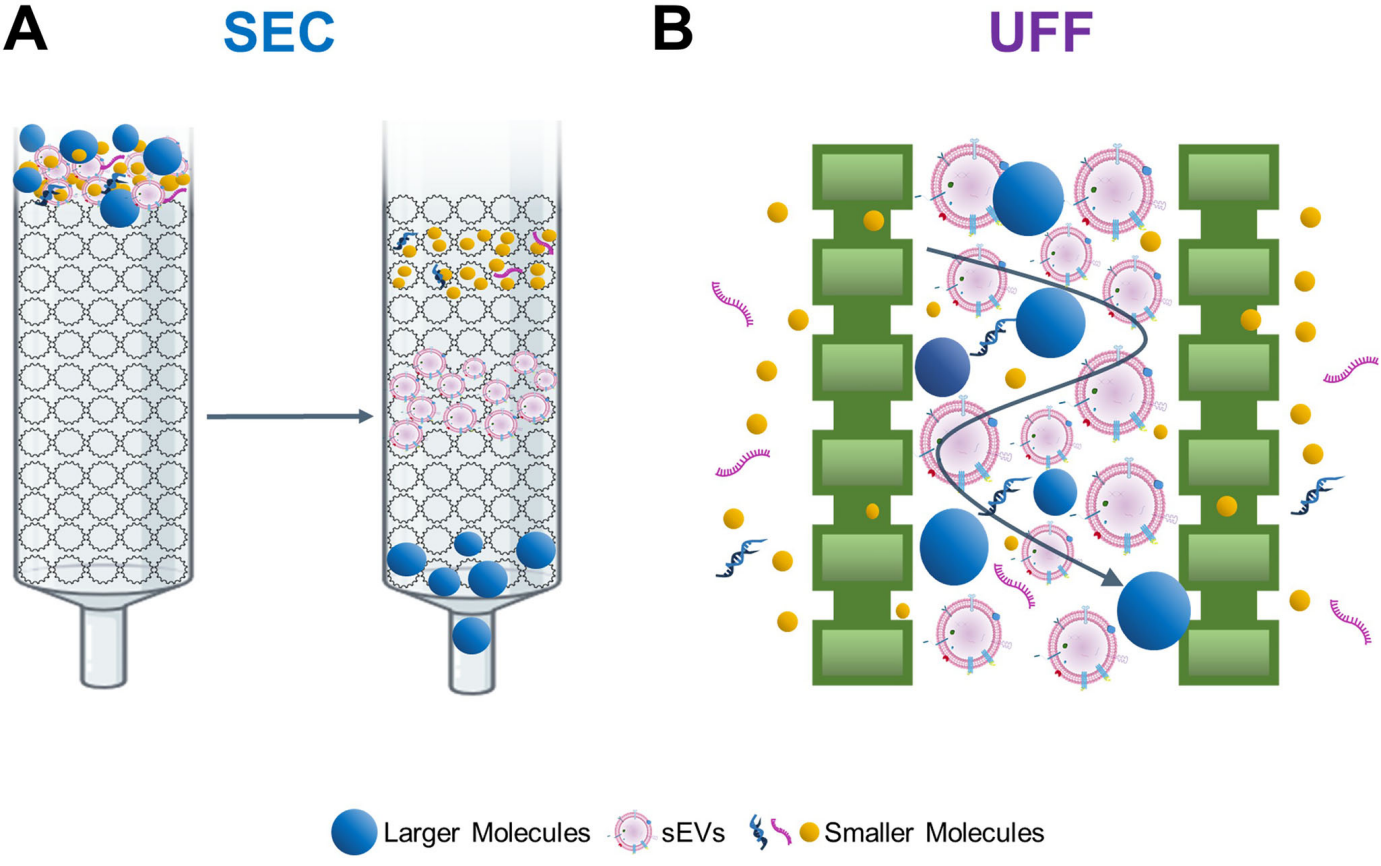
**The methodological principle of SEC and UFF. (A)** SEC columns consist of a porous resin material, allowing for the separation of molecules based on size. Using gravity‐based flow, molecules smaller than the pore size enter the pores, slowing their elution, whereas larger molecules cannot enter the pores, and therefore elute faster. This results in the enrichment of sEVs in specific flow‐through fractions. **(B)** The UFF device consists of a dual‐filter reservoir connected to a nanoporous membrane. Application of membrane vibrations and negative pressure oscillations cause molecules smaller than the filter size to be removed and molecules larger than the filter size to be retained. This results in the enrichment of sEVs in the sample reservoir. SEC: Size Exclusion Chromatography; UFF: Ultrafast Filtration.

To assess the impact of different isolation techniques on sEV protein cargo, sEVs were isolated from the plasma of healthy individuals. We previously demonstrated that size exclusion chromatography (SEC; Welsh et al. 2024) is an effective method for the isolation of plasma sEVs (Lobb et al. 2015), due to its gentle nature, enrichment of sEV markers, capacity to reduce albumin and produce high particle‐to‐protein ratios (Lobb et al. 2015; Gámez‐Valero et al. 2016; Kong et al. 2025; Brennan et al. 2020). SEC columns consist of porous resin that separates particles according to hydrodynamic size. Thus, retention time correlates with particle size, resulting in the elution of sEVs, separate from smaller or larger molecules (Clos‐Sansalvador et al. 2022) (Figure 1A). However, the relatively low particle recovery of SEC is unfavourable in large‐scale clinical settings (Sidhom et al. 2020; Turner et al. 2022). An automated ultrafast filtration (UFF; Srinivasan et al. 2019) system has recently emerged on the market and has been reported to be time‐efficient, produce high sEV yields, high particle‐to‐protein ratios, enrich sEV markers and reduce albumin (Ye et al. 2022). This UFF device uses a nanoporous anodic aluminium oxide membrane that is connected to both sides of a dual‐filter reservoir. Alternation between negative pressure oscillations and membrane vibrations result in the elimination of small particles (e.g. nucleic acids), retention of sEV‐sized particles and resuspension of particles within the reservoir to prevent membrane fouling (Chen et al. 2021) (Figure 1B).

Hence for the isolation of plasma sEVs, SEC and UFF were utilised, and the particle and protein yields, and proteomic profiles were assessed to determine similarities and differences of the resulting sEV preparations. Overcoming challenges in isolating appropriate sEV populations is critical for improving the reliability and efficacy of sEV‐based applications. High‐quality sEV preparations enable robust diagnostic and analytical performance and enhance the reproducibility of research findings (Busatto et al. 2018). The selection of an appropriate isolation method is therefore essential to ensure that results accurately reflect EV compositions and support meaningful downstream interpretation.

## 2 Materials and Methods

### 2.1 Blood Collection and Plasma Preparation

Healthy subjects (*n* = 20) were recruited from the Department of Otorhinolaryngology, Head and Neck Surgery of the Prince of Wales Hospital, Hong Kong. Informed consent was given by all donors involved in this study, and the study was approved by The Joint Chinese University of Hong Kong–Hospital Authority New Territories East Cluster Clinical Research Ethics Committee (2015.396). Whole blood was collected in EDTA‐coated tubes before plasma processing. The whole blood was centrifuged at 1600 x *g* at 4°C for 10 min. The resulting plasma supernatant was transferred to a new tube and centrifuged at 1600 × *g*, at 4°C for 10 min, to eliminate potential cellular contaminants. Plasma was snap frozen on dry ice and stored as 2 mL aliquots at −80°C. Before sEV isolation, plasma was thawed on ice and plasma from two different individuals were pooled at random, giving a total of ten plasma pools. The plasma pools were centrifuged at 10,000 × *g* at 4°C for 20 min to remove large vesicles. Equal volumes of pooled plasma (430 µL) were utilised for each EV isolation method employed (ultracentrifugation, size exclusion chromatography and ultrafast filtration).

### 2.2 Ultracentrifugation

Isolation of sEVs from plasma was performed using a Beckman Coulter Optima XPN‐100 Ultracentrifuge (50.2 Ti rotor). The plasma (430 µL) was diluted in PBS (1:10) and centrifuged at 100,000 × *g*, at 4°C for 2 h to pellet the sEVs. The supernatant was carefully discarded and the crude sEV pellets were rinsed with 1 mL ice‐cold PBS and centrifuged at 100,000 × *g*, at 4°C for 2 h. The resulting sEV pellets were resuspended in 500 µL PBS and stored at −80°C.

### 2.3 Size Exclusion Chromatography

SEC columns (qEVoriginal 70 nm, IZON) were utilised for sEV isolation as previously reported (Lobb et al. 2015). Briefly, a total volume of 430 µL plasma was diluted in PBS (up to 500 µL) and overlaid on the column (fraction 1), followed by five 400 µL PBS fractions (fractions 2–6), which were discarded. The sEV‐enriched fractions (fractions 7–12) were collected (a total volume of 2400 µL) and concentrated in Amicon Ultra‐4 10 kDa centrifugal filter units (Merck Millipore) at 4000 × *g*, at 4°C (Beckman Coulter Allegra V‐15R Benchtop Centrifuge) to a final volume of approximately 200 µL.

### 2.4 Ultrafast Filtration

A medium (30 nm) exosome isolation device (Talebjedi et al. 2021) was used for the isolation of sEVs from plasma, in the EXODUS H600 Automatic Exosome Isolation system (HUIXIN LIFETECH). Plasma (430 µL) was diluted in PBS (1:60) and filtered through a 0.22 µm filter (Millipore). A built‐in optimised isolation protocol (Plasma.Strong‐MA.A03 mode) was employed, which included settings of negative pressure at −30 kPa, resonant frequency at 6,250 Hz, pressure switching time at 10 s, pressure switching interval time at 5 s. After isolation, 400 µL PBS was added into the EID to thoroughly rinse both sides of the membrane to collect the sEVs.

### 2.5 Nanoparticle Tracking Analysis

Quantification and size profiling of sEVs was measured using ZetaView (Particle Metrix, Germany), software ZetaView (version 8.05.16 SP7). Various specifications were set, including working temperature: 25°C; pH: 7.0; laser wavelength: 488 nm; filter wavelength: scatter; sensitivity: 80.0; shutter: 100; size distribution acquired 1 cycle in 11 positions; max area 1000; min area 10; min brightness 20; classes/decade: 64. According to the manufacturer's instructions, 100 nm polystyrene particles (1.8 × 10^13^ particles/ml) were diluted in PBS (1:250000) were used to standardise particle size and concentration for auto‐alignment. The sEV samples were diluted to 1 mL in PBS, and carefully injected by syringe into the sample chamber, to avoid air bubbles and drifting. Samples were analysed in triplicate. Quantification of particle yields, and size distribution was normalised to the starting volume of plasma, as well as the final volume of sEV sample.

### 2.6 Transmission Electron Microscopy

The sEVs were fixed in 2% paraformaldehyde, 10 µL fixed sEV samples were loaded on 200‐mesh carbon‐coated copper grid for 5 min, negatively stained with 10 µL 4% uranyl acetate total 2 min, washed with filtered distilled water, dried on filtered paper for 2 h. The sEV samples were visualized using a Hitachi HT7700 Transmission Electron Microscope at 100 kV.

### 2.7 Total Lipid Quantification

Total lipid content of sEVs was measured by sulfo‐phospho‐vanillin (SPV) assay, as previously described (Visnovitz et al. 2019; Bodnár et al. 2025). To increase the sensitivity, 1 mg/ mL unsaturated 1,2‐Dioleoyl‐sn‐glycero‐3‐phosphocholine (DOPC) (P6354, Sigma Aldrich) liposome stock solution was dissolved in chloroform (C2432‐1L, Sigma Aldrich). PBS was added to the dry DOPC after evaporating the chloroform at 60°C dry bath under a fume hood. Standards (with serial dilutions ranging from 0 to 1 µg/ µL, in PBS) and sEV samples (1:8 dilution in PBS) were prepared, followed by 1:6 dilution in 96% sulphuric acid (M100731.2500, Sigma Aldrich). The standards and sEV samples were incubated at 90°C for 20 min, cooled down to room temperature, followed by addition of 120 µL of freshly prepared phosphor‐vanillin reagent (50 mg of vanillin (V1104, Sigma Aldrich) dissolved in 50 mL of 17% phosphoric acid (345245‐100 M, Sigma Aldrich)). A final volume of 280 µL of the standards and sEV samples were transferred into a non‐treated 96‐well microplate and incubated at 37°C for 1 h with shaking. Absorbance was measured at 540 nm (BioTek SYNERGY H1 microplate reader (Software Gen5 3.12)).

### 2.8 RNA Isolation

RNA extraction was performed using equal particle numbers (3 × 10^11^) isolated by SEC (*n* = 5) and UFF (*n* = 5), according to the manufacturer's protocol (75144, Qiagen). Briefly, 3 × 10^11^ particles were 1:3 dilution in Trizol Reagent (15596018, Thermo Fisher) and incubated at room temperature for 5 min. To achieve phase separation, chloroform (80 µL) (C2432‐1L, Sigma Aldrich) was added, the mixture was inverted several times, incubated at room temperature for 2 min, then centrifuged at 12,000 × *g* for 15 min at 4°C. The upper aqueous phase was transferred to a new collection tube and mixed with 200 µL ethanol. The final volumes were added to a RNeasy Mini column and centrifuged at ≥ 8000 × *g* for 15 s at room temperature. The column washed with buffer three times. To elute the RNA, 20 µL RNase‐free water was added to the RNeasy Mini column then centrifuged at ≥ 8000 × *g* for 1 min. RNA quality, yield, and size was assessed using the Agilent 2100 Bioanalyzer (Agilent Technologies) using the Agilent Pico RNA chip.

### 2.9 Next‐Generation Sequencing

Library of small RNA was prepared using Small RNA Lib Prep Kit for Illumina library (RK20312, ABclonal). RNA 3' Adapter (RA3), 5'‐AGATCGGAAGAGCACACGTCT‐3 ‘ and RNA 5’ Adapter (RA5), 5’‐GTTCAGAGTTCTACAGTCCGACGATC‐3' are ligated to 3’ and 5’ end of small RNA, respectively, followed by a reverse transcription reaction and PCR amplification and enrichment. Libraries with insert fragments ranging from 18 to 40 base pairs were sequenced using the Illumina sequencing platform NovaSeq6000 SP Reagent kit V1.5, using SE50 read length. Read counts were normalized by the trimmed mean of M values (TMM) method with the edgeR package in R (Robinson et al. 2010). Define the miRNAs detectable in all five samples within each group.

### 2.10 Proteinase K Treatment

Proteinase K digestion was conducted as previously reported (Lima et al. 2021). Briefly, 4 µg plasma and sEVs were treated with 0.5, 1, 2.5, 5, 10 µg/mL proteinase K at 37°C for 5 min (P2308, Sigma–Aldrich), and the proteinase activity was then inactivated by incubation at 90°C for 5 min.

### 2.11 Protein Quantification and Western Blot Analysis

Protein concentrations of plasma and sEVs were measured using Bradford reagent (5000205, Bio‐Rad) and quantified by BioTek SYNERGY H1 microplate reader (Software Gen5 3.12). Western blots were performed as previously described (Visan et al. 2022). Briefly, plasma and sEVs were lysed in reducing (0.278 M Tris‐ HCl (pH 6.8), 44.4% glycerol, 4.4% SDS, 0.355 M 2‐mercaptoethanol and 0.02% bromophenol blue) or non‐reducing (without 2‐mercaptoethanol) buffer. Either equal particle (5.0 × 10^9^ particles) or equal protein (5.0 µg) amounts were loaded. Proteins were resolved and separated on SDS‐polyacrylamide gels (4% stacking gel and 10% resolving gel) and transferred onto PVDF membranes using Trans‐Blot Turbo Transfer System (Bio‐Rad). Membranes were blocked with 10% non‐fat milk in PBS‐T (0.5% Tween‐20) for 1 h and incubated with primary antibodies at 4°C overnight. Antibodies included mouse anti‐Albumin (1:1000) (MA5‐50835, Thermofisher), mouse anti‐Flotillin‐1 (1:1000) (610821, BD Biosciences), mouse anti‐HSP70 (1:1000) (610608, BD Biosciences), mouse anti‐TSG101(1:1000) (sc7964, Santa Cruz), mouse anti‐CD81 (1:1000) (SC166029, Santa Cruz), rabbit anti‐CD9 (13174S, Cell Signaling Technology), mouse anti‐ApoA1 (1:1000) (MIA1404, Thermofisher), mouse anti‐APOE (1:3000) (66830‐1‐Ig, Proteintech), rabbit anti‐IgG4(Fc) (1:1000) (A23201, ABclonal). Subsequently, the membranes were washed with PBS‐T and incubated with secondary antibodies, anti‐mouse IgG, HRP (1:30,000) (7076S, Cell Signaling Technology) or anti‐rabbit IgG, HRP (1:10,000) (7074S, Cell Signaling Technology) for 1 h at room temperature. Proteins were visualised using ECL Prime or Select Western Blotting Detection Reagent (GERPN2236; GERPN2235, Merck) and imaged using the software Compass for SW.

### 2.12 Capillary Western Blot (Jess) Analysis

Capillary Western blot analysis was performed using the ProteinSimple Jess apparatus (ProteinSimple, Bio‐Techne). Plasma and sEVs were denatured using 5× Fluorescent Master Mix, boiled at 95°C for 5 min, then loaded into Wes multi‐well plates. The following primary antibodies were used: mouse anti‐Albumin (1:100; MA5‐50835, Thermofisher), mouse anti‐ApoA1 (1:500; MIA1404, Thermofisher), mouse anti‐ APOE (1:500; 66830‐1‐Ig, Proteintech), mouse anti‐CD81 (1:20; SC166029, Santa Cruz), mouse anti‐Flotillin‐1 (1:50; 610821, BD Biosciences), mouse anti‐HSP70 (1:5; 610608, BD Biosciences), mouse anti‐TSG101 (1:20; sc7964, Santa Cruz), mouse anti‐Syntenin‐1 (1:100; SC515338, Santa Cruz), rabbit anti‐CD63 (1:20; sc‐15363, Santa Cruz), rabbit anti‐Complement C3 (1:500; ab200999, Abcam), mouse anti‐Alix (1:50; sc‐53540, Santa Cruz), mouse anti‐Annexin 1 (1:50; sc‐12740, Santa Cruz). Secondary antibodies, anti‐mouse IgG, HRP (1:30,000; 7076S, Cell Signaling Technology) or anti‐rabbit IgG, HRP (1:10,000; 7074S, Cell Signaling Technology) were used according to the manufacturer's instructions. Images were captured and analysed using Compass software for Simple Western (ProteinSimple, Bio‐Techne) with 12–230 kDa Separation Module.

### 2.13 Enzyme‐Linked Immunosorbent Assay (ELISA)

Abundance of complement component C3, MAC‐2BP, CD9, CD63, CD81 were measured using Human Complement C3 ELISA Kit (ab108823, Abcam), Human Galectin‐3BP/MAC‐2BP DuoSet ELISA (DY2226, R&D Systems), ExoELISA‐ULTRA Complete Kit (CD9 detection) (EXEL‐ULTRACD9‐ 1, System Biosciences), ExoELISA‐ULTRA Complete Kit (CD63 detection) (EXEL‐ULTRACD63‐ 1, System Biosciences), ExoELISA‐ULTRA Complete Kit (CD81 detection) (EXEL‐ULTRACD81‐ 1, System Biosciences), according to the manufacturer's instructions.

### 2.14 Sample Preparation for Proteomic Analysis

Plasma‐derived sEVs (20 µg) isolated by SEC (*n* = 8) or UFF (*n* = 8) were prepared in EasyPep lysis buffer (Thermofisher). Protein was reduced and alkylated at 95°C for 10 min, then cooled to room temperature. Digestion was carried out by addition of LysC / trypsin for 180 min at 37°C. Finally, digestion was stopped by acidification and peptides were washed and desalted using the spin column provided in the Easypep kit (Thermofisher).

### 2.15 Data‐Independent Acquisition Quantitation Analysis

Peptides were loaded and separated over with commercial C18 column (75 µm i.d. × 50 cm length × 2 µm) coupled to a NanoTrap column (75 µm i.d. × 2 cm length × 3 µm) (Thermo Fisher). Dionex Ultimate3000 nanoRSLC system coupled to Thermo Fisher Orbitrap Fusion Tribid Lumos mass spectrometer. Trapping was conducted under a linear gradient of increasing buffer B (80% ACN and 0.1% FA) and declining buffer (0.1% FA) at 300 nL/ min. Buffer B (to 30% B, from 0–164 min, and 30%–44% B, from 164–214 min, 44%–80% B, from 214–221 min, followed a quick ramp back to 5% B). A data‐independent (DIA) method was used for peptide acquisition. MS1 resolution: 120k, AGC target: 250%, maximum ion injection time (IT): 45 ms. MS2 isolation window 75 × 14 Da windows from 350 to 1400 m/z with a 1 Da overlap on each side. Raw mass spectrometry data were conducted using Spectronaut (BIOGNOSYS). MS analysis was performed on Spectronaut v.14 (BIOGNOSYS) using default settings without N‐acetyl variable modification enabled. A discovery rate of 1% was used for protein level and PSM, respectively; respectively. Data filtering was set to Q‐value and normalization set to global normalization.

### 2.16 Statistical Analysis

GraphPad Prism 10.2.3 (GraphPad Software) was used for statistical analyses. All experiments were conducted by two different individuals and a minimum of three independent times. The values are presented as mean ± standard error (S.E.M.). Paired *t*‐test was used for statistical analysis of all data. A *p*‐value less than 0.05 was deemed as significantly different, *p < 0.05, **p < 0.01.

## 3 Results

### 3.1 UFF Isolates Higher Concentrations of Particles and Protein, but SEC Isolates Larger Particles With Higher Particle‐To‐Protein Ratios

**FIGURE 2:**
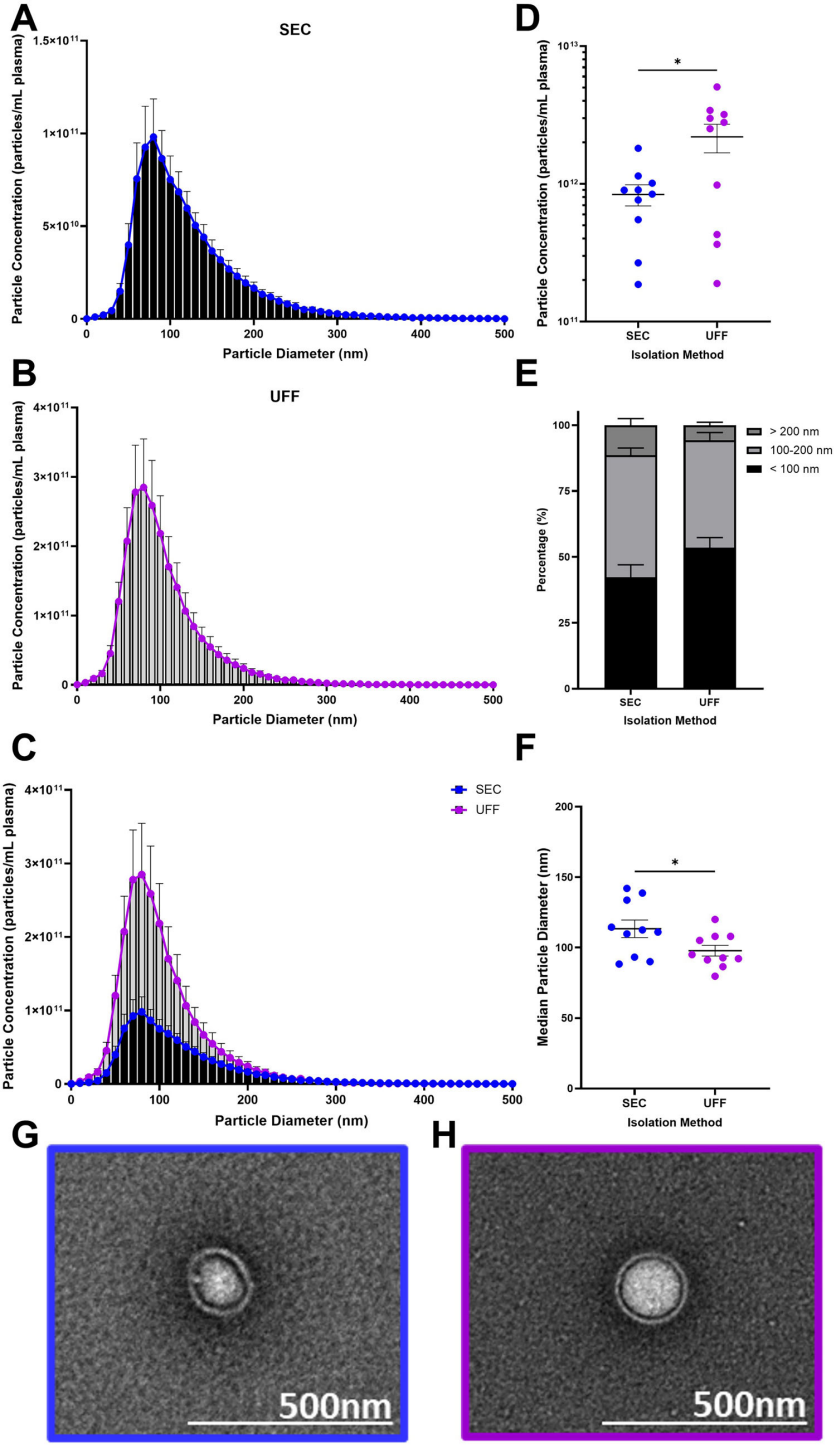
**SEC isolates larger particle sizes, whilst UFF isolates higher particle yields from human plasma. (A–C)** Size distribution, **(D)** particle concentration, **(E)** percentage of particle size ranges and **(F)** median particle diameter (nm) were assessed by nanoparticle tracking analysis. Representative transmission electron microscopy images demonstrate a round, cup‐shaped morphology of particles isolated by **(G)** SEC and **(H)** UFF. Size bar is 500 nm. Data are presented as *n* = 10±SEM. *p <0.05. Statistical analyses were performed using paired *t*‐test, Wilcoxon test. SEC: Size Exclusion Chromatography; UFF: Ultrafast Filtration.

To determine sEV yield, equal volumes of the same pooled human plasma (430 µL) were used as input material. Of note, the same plasma sample was processed in parallel by SEC and UFF (Figure 1), allowing direct comparisons. Utilising UC (Figure S1), SEC and UFF (Figure 2), sEVs were isolated from plasma, and the size distribution profiles and particle concentrations were measured using nanoparticle tracking analysis (Figure 2A–D, Figure S1B,C). Although UC effectively isolated particles within the sEV size range (Figure S1D–G), UC produced inconsistent and relatively low particle and protein yields (Figure S1B,C,H), resulting in suboptimal ratios of particles per microgram of protein (Figure S1I). These results determined that UC was unsuitable for sEV isolation for use in downstream clinical applications, hence UC was not further investigated in this project.

Both SEC and UFF isolated particles within the sEV size range, however, UFF isolated a significantly higher number of particles compared to SEC (Figure 2A–D, Figure S2A,B). Interestingly, SEC isolates a significantly higher percentage of larger EVs (> 200 nm), in comparison to UFF (Figure 2E, Figure S2C–E), resulting in significantly larger median particle sizes (Figure 2F). The size distribution is consistent with the separation characteristics of the 70 nm SEC column used, noting that SEC performance is inherently dependent on column pore size and material (Potakowskyj et al. 2025). Regardless, both methods result in the enrichment of particles that resemble a typical, cup‐shaped morphology as imaged by transmission electron microscopy, although the background signal predominantly consisted of non‐EV particles (Figure 2G,H, Figure S2F,G).

**FIGURE 3:**
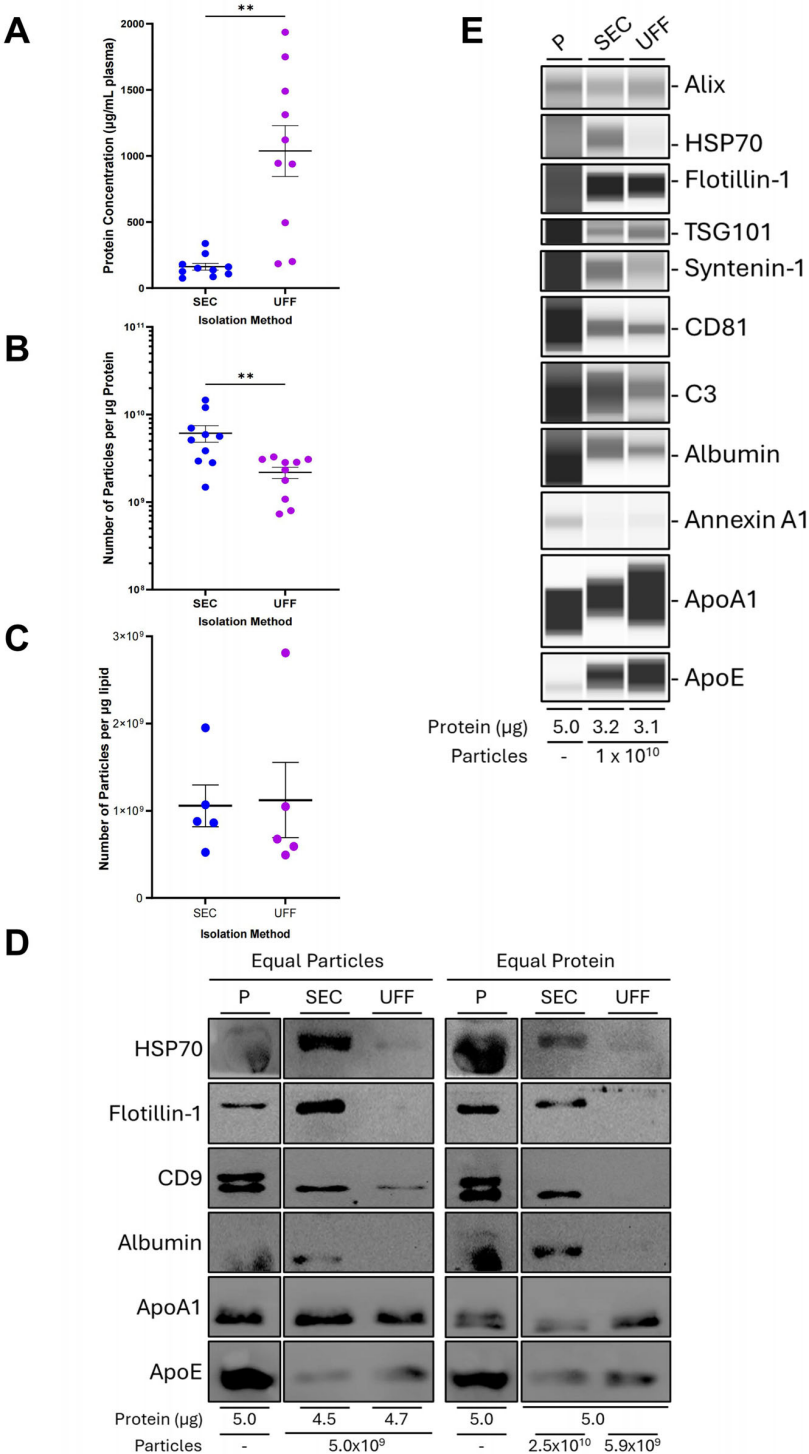
**UFF recovers higher protein yields; however, SEC recovers particles with higher particle‐to‐protein ratios and an increased abundance of characteristic EV markers. (A)** Protein concentration measured by Bradford Assay. **(B)** Number of particles per microgram of protein. **(C)** Number of particles per microgram lipid. **(D)** Western blot analyses of EV protein markers HSP70, Flotillin‐1 and CD9 in plasma (P) and EVs isolated by SEC and UFF. The serum protein Albumin and apolipoproteins ApoA1 and ApoE, were used to assess the presence of secreted proteins typically recovered with blood‐derived EVs. Equal particle numbers (5.0 × 10^9^ particles) and equal protein amounts (5.0 µg) of EVs were loaded. The corresponding protein and particle amounts are shown. **(E)** Capillary‐based western blot analyses of EV protein markers Alix, HSP70, Flotillin‐1, TSG101, Syntenin‐1 and CD81 in plasma (P) and EVs isolated by SEC and UFF. The exocytosis protein Annexin A1 and serum proteins C3, Albumin, ApoA1 and ApoE, were used to assess the presence of large EVs and secreted proteins typically recovered with blood‐derived EVs. Equal particle numbers (1.0 × 10^10^ particles) were loaded. The corresponding protein amounts are shown. Data are presented as *n* = 10±SEM. **p <0.01. Statistical analyses were performed using paired t‐test, Wilcoxon test. SEC: Size Exclusion Chromatography; UFF: Ultrafast Filtration.

The ability of UFF to produce high particle yields is also reflected by the high protein concentrations (Figure 3A). Although UFF sEVs have significantly higher protein abundance compared to SEC sEVs, the protein yields cover a relatively broad concentration range (Figure 3A). As a result, SEC sEVs have significantly higher ratios of particles per microgram of protein (Figure 3B), a measurement erroneously referred to as purity, but indicative of elevated particle abundance and reduced non‐EV protein content. Interestingly, sEVs isolated by SEC and UFF have comparable particle‐to‐lipid ratios, a measurement suggestive of high particle counts and low non‐EV lipids (Figure 3C).

### 3.2 Assessment of the Presence of EV and Non‐EV Proteins Reveals a Higher Abundance of EV‐Associated Proteins in SEC sEVs

To evaluate particle‐specific protein abundance, western blot analyses was conducted with equal particle and equal protein loading (Figure 3D). Surprisingly, in equal particle loading, the EV marker Flotillin‐1 was only detected in SEC sEVs, and the EV markers HSP70 and CD9 were detected at a much higher abundance in SEC sEVs, compared to UFF sEVs (Figure 3D). Some of the most abundant circulating proteins in serum were assessed for their presence in both isolations. Albumin was only detected in SEC sEVs, whereas apolipoproteins ApoA1 and ApoE were detected in both SEC and UFF sEVs (Figure 3D). Similarly to data from analysing equal particle loading, equal protein loading revealed that HSP70 was detected at a higher abundance in SEC sEVs, whilst Flotillin‐1 and CD9 were only detected in SEC sEVs. Again, Albumin was only detected in SEC sEVs, and ApoA1 and ApoE were detected in both SEC and UFF sEV preparations (Figure 3D).

The only differing factor in the final sEV products was the methodology utilised for particle isolation. Despite this, most of the sEVs isolated from the same plasma sources had contrasting particle and protein concentrations (Table S1). As a result, the abundance of sEV markers varied greatly (Figure S3A,B), creating difficulty for appropriate comparisons. Notably, Flotillin‐1 was detected at a similar abundance in both SEC and UFF sEVs, despite SEC sEVs having a 16.6‐fold lower protein loading. On the other hand, HSP70 and TSG101 were detected at a similar abundance in both SEC and UFF sEVs, despite UFF sEVs having a 4.9‐fold lower particle loading (Figure S3A).

As an additional measure to assess protein presence, a highly sensitive, automated, capillary‐based Western blot system was utilised. Assessment of equal particle numbers revealed comparable abundance of EV markers Alix, Flotillin‐1, TSG101 and CD81 in SEC and UFF sEVs, whereas HSP70 and Syntenin‐1 were only detected in SEC sEVs (Figure 3E). Both Albumin and Complement protein C3, were detected at a higher abundance in SEC sEVs, whereas the exocytosis marker Annexin A1, and apolipoproteins ApoA1 and ApoE were enriched in UFF sEVs (Figure 3E). Increased abundance of HSP70 in SEC sEVs, and ApoA1 and ApoE in UFF sEVs was consistent across other sEV preparations, however, the abundance of other proteins varied greatly (Figure S3B). To remove non‐EV‐associated proteins, sEVs were treated with various concentrations of a protease, proteinase K. With increasing proteinase K concentration, signals for immunoglobulin IgG and apolipoprotein ApoE progressively diminished, indicating proteinase sensitivity. At elevated proteinase K concentrations, the EV marker CD81 demonstrated similar concentration‐dependent signal weakening, consistent with characteristics of excessive digestion and/or degradation. These data indicate that the immunoglobulin detected in the sEV preparations were not protease‐protected, and thus likely represents surface‐accessible proteins or co‐isolated proteins rather than luminal sEV cargo (Figure S3C).

### 3.3 Next‐Generation Sequencing and Mass Spectrometry Revealed the Enrichment of microRNAs and Transmembrane Proteins in SEC and UFF sEVs

**FIGURE 4:**
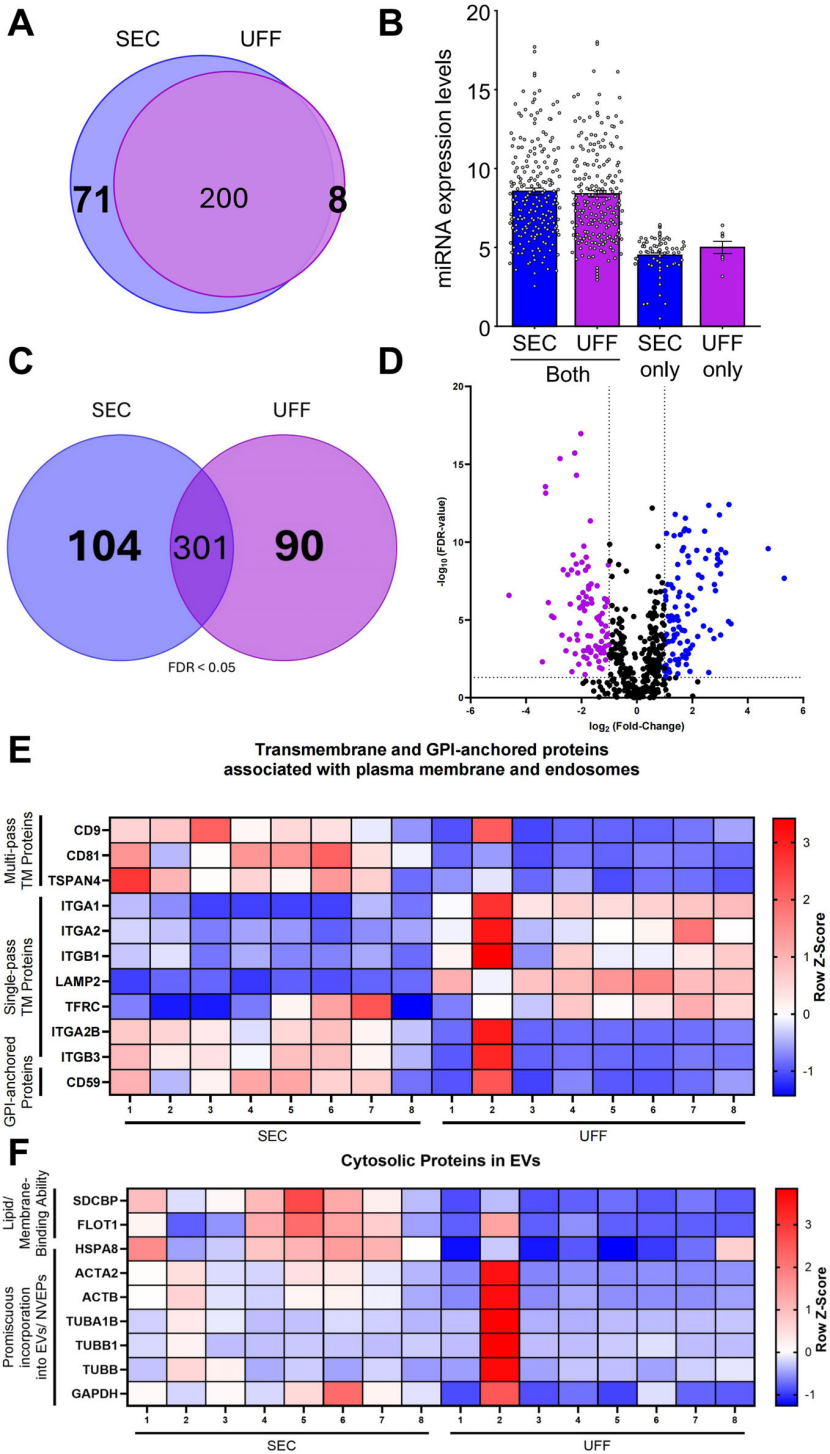
**SEC and UFF isolate particles from human plasma with key proteomic differences in the abundance of EV‐associated proteins. (A)** Venn diagram and **(B)** Bar chart of miRNAs detected across all five samples per group. The diagram illustrates the overlap of miRNAs consistently detected in all five samples within the SEC and UFF groups. A total of 200 miRNAs were commonly detected in both groups, while 71 and 8 miRNAs were unique to SEC and UFF, respectively. **(C)** Venn diagram and **(D)** Volcano plot of 495 proteins commonly identified in both SEC and UFF EVs, as determined by label‐free mass spectrometry. The horizontal black dotted line corresponds with a significant FDR p‐value <0.05 and the vertical black dotted line corresponds with a fold‐change larger than 2‐fold. Significantly enriched proteins in SEC and UFF EVs are represented as blue and purple dots, respectively. Heatmaps of mass spectrometry data analysing abundances of EV‐associated **(E)** transmembrane and GPI‐anchored proteins associated with plasma membrane and endosomes and **(F)** cytosolic proteins. The colour key denotes the row z‐score. Data are presented as *n* = 8. NVEPs: non‐vesicular extracellular particles; SEC: Size Exclusion Chromatography; TM: transmembrane: UFF: Ultrafast Filtration. *Note: the full list of miRNAs detected across all five samples per group is provided in Table S2.

To quantify microRNA (miRNA) expression levels in sEVs, next‐generation sequencing was conducted, detecting a total of 279 miRNAs (Figure 4A). Of these, 200 miRNAs were detected in both SEC and UFF sEVs, however, 71 miRNAs were detected exclusively in SEC sEVs, whereas 8 were unique to UFF sEVs (Figure 4A,B, Table S2). To assess methodological influences on miRNA content, correlation analyses were performed. The five sample pairs exhibited correlations (Figure S4A), indicating similarity between sEVs isolated using SEC and UFF.

Protein‐based assessments have been widely accepted as the primary means of sEV characterisation (Lobb et al. 2017; Wen et al. 2019). To evaluate the proteomic profiles of sEVs isolated by SEC and UFF, label‐free mass spectrometry analysis was performed. A statistically significant threshold was established with a minimum 2‐fold difference in abundance and *p*‐value of less than 0.05. Mass spectrometry analysis detected a total of 495 proteins, with 104 and 90 proteins significantly enriched in SEC and UFF sEVs, respectively (Figure 4C,D). The Minimal Information for Studies of Extracellular Vesicles 2023 (MISEV2023) guidelines provide an extensive list of EV and non‐EV‐associated proteins (Welsh et al. 2024). Hence, to ensure that the particles isolated by SEC and UFF were truly representative of sEVs, various proteins were investigated for their abundance. The most widely recognised sEV markers are the multi‐pass tetraspanin proteins CD9, CD81 and CD63 (Fan et al. 2023; Jeppesen et al. 2019; Andreu and Yáñez‐Mó 2014). Mass spectrometry revealed that both CD9 and CD81 were significantly enriched in SEC sEVs compared to UFF sEVs (Figure 4E), however, CD63 was not detected (Table S3). The increased abundance of CD9‐positive particles in SEC sEVs was also demonstrated by ELISA (Figure S5A). However, ELISA‐based measurements showed no significant difference between SEC and UFF sEVs in the number of CD81‐positive particles (Figure S5B). Interestingly, CD63 was quantifiable by ELISA, however, at relatively low levels and with no significant difference between SEC and UFF sEVs (Figure S5C).

**FIGURE 5:**
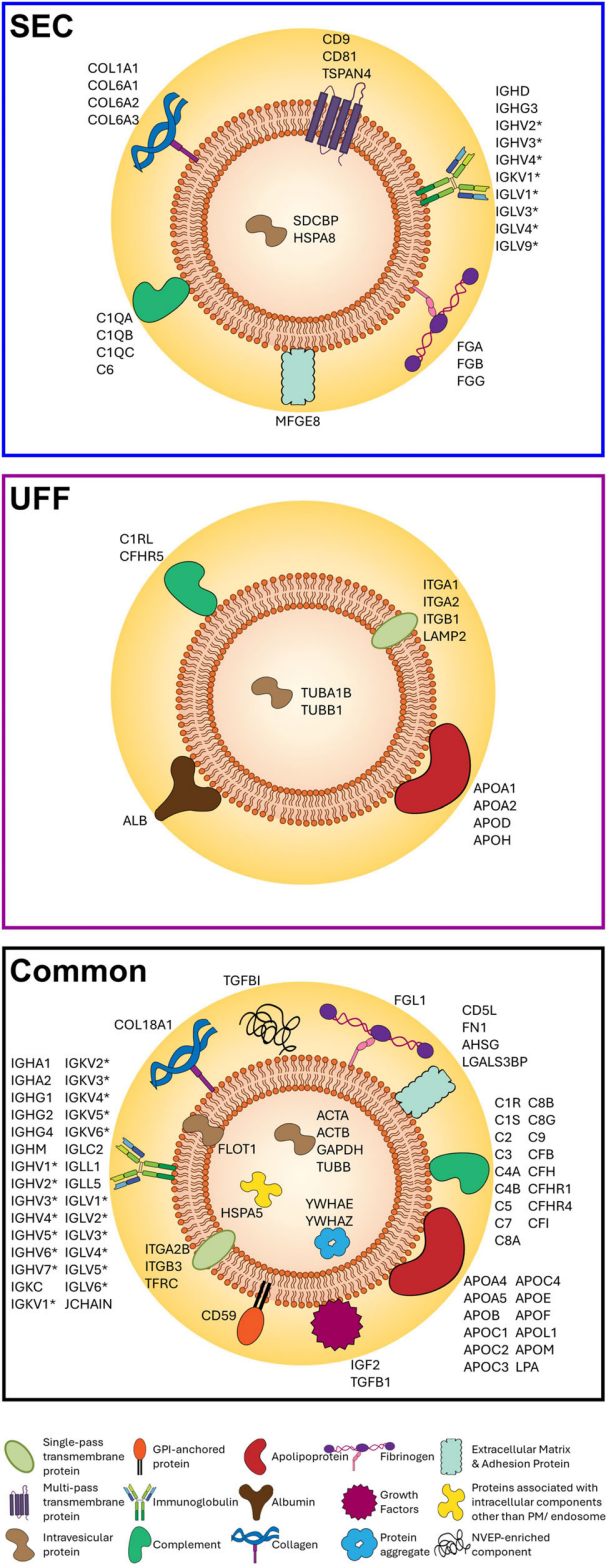
**A graphical summary of EV‐ and non‐EV‐ associated proteins significantly enriched in sEVs isolated by SEC and UFF, as well as those of comparable abundance, as identified by mass spectrometry**. SEC and UFF sEVs are denoted by the blue and purple outlined box, respectively. Proteins of comparable abundance between SEC and UFF sEVs are denoted by the black outlined box. *Note: The immunoglobulin proteins listed in this figure are summarised, the full list of proteins is provided in Table S3.

Other EV‐associated proteins amongst the 104 enriched in SEC sEVs were multi‐pass transmembrane marker TSPAN4 (Figure 4E, Figure 5). Amongst the 90 proteins enriched in UFF sEVs were single‐pass transmembrane proteins LAMP2 and integrins ITGA1, ITGA2 and ITGB1 (Figure 4E, Figure 5). GPI‐anchored protein CD59 and single‐pass transmembrane proteins TFR1, ITGA2B and ITGB3 were present in both SEC and UFF sEV preparations. Proteins of cytosolic origin are often packaged into EVs (Doyle and MZ. 2019; Gurung et al. 2021). Of the cytosolic proteins detected, Syntenin‐1 (SDCBP) and heat shock protein HSPA8 were enriched in SEC sEVs and tubulin proteins TUBA1B and TUBB1 were enriched in UFF sEVs (Figure 4F, Figure 5). The canonical sEV markers Flotillin‐1 (FLOT1) and GAPDH, as well as actins ACTA and ACTB and tubulin TUBB, were of comparable abundance in SEC and UFF sEVs. Of note, although not statistically significant, traditional EV marker Flotillin‐1 was detected in seven of the eight SEC sEV samples, but only in two of the eight UFF sEV samples (Figure 4F, Figure 5, Table S3).

### 3.4 SEC Isolates Particles Highly Abundant in Immunoglobulin, Complement and Fibrinogen Proteins, Whilst UFF Isolates Particles Highly Abundant in Apolipoproteins and Albumin

Not only is it essential that EV protein markers are detected, but it is also crucial to evaluate the presence of non‐EV proteins, due to the potential impact on downstream applications (Konoshenko et al. 2018; Janouskova et al. 2022). MISEV2023 categorises proteins that are not of EV origin, but often co‐isolated together with EVs (Welsh et al. 2024). MISEV2023 categorised Albumin, lipoproteins, immunoglobulins and NVEP‐enriched proteins as ‘major components of non‐EV co‐isolated structures’ (Welsh et al. 2024). Albumin was detected at a significantly higher abundance in UFF sEVs, compared to SEC sEVs (Figure 5, Figure S6), varying from findings of traditional and capillary‐based western blot analyses (Figure 3C,D, Figure S3B). Interestingly, a total of 111 immunoglobulins were detected overall, with 13 immunoglobulins enriched in SEC sEVs and zero immunoglobulins enriched in UFF sEVs (Figure 5, Figure S7). For NVEP‐associated proteins, TGFBI, YWHAE and YWHAZ were of similar abundance between SEC and UFF sEVs (Figure 5, Figure S6). Mass spectrometry revealed a significant increase of numerous apolipoproteins in UFF sEVs compared to SEC sEVs, including APOA1, APOA2, APOD and APOH (Figure 5, Figure S6). Notably, the enrichment of APOA1 in UFF sEVs (Figure 5, Figure S6), validates the findings of traditional and capillary‐based western blot analyses (Figure 3C,D, Figure S3B). According to MISEV2023, not only are lipoproteins classified as non‐EV co‐isolated structures, but they are also considered as blood‐derived EV corona proteins.

Blood‐derived EV corona proteins also include complement and fibrinogen proteins (Welsh et al. 2024). Numerous complement proteins were detected, with a significant enrichment of C1Q (A/B/C) and C6 in SEC sEVs, and C1RL and CFHR5 in UFF sEVs (Figure 5, Figure S8A). Comparable abundances of most of the complement proteins, including C3, were detected between SEC and UFF sEVs (Figure 5, Figure S8A). This was also confirmed by ELISA, showing similar abundance of C3 between SEC and UFF sEVs (Figure S8B). Four fibrinogen proteins were detected, with three of the four, FGA, FGB and FGG, significantly higher in SEC sEVs (Figure 5, Figure S8A).

Other secreted proteins recovered with EVs include growth factors and adhesion and extracellular matrix proteins (Welsh et al. 2024). There was no difference in the abundance of growth factors TGFB1 and IGF2 (Figure 5, Figure S8A). SEC and UFF sEVs contain various adhesion and extracellular matrix proteins, with similar abundance of FN1, AHSG, LGALS3BP, CD5L and COL18A1. SEC sEVs contain significantly higher amounts of MFGE8 and collagen proteins COL1A1, COL6A1, COL6A2 and COL6A3 (Figure 5).

As sEVs are of endosomal and plasma membrane origin, proteins associated with other intracellular compartments should not be present. However, an endoplasmic reticulum protein HSPA5 was detected in comparable abundance between SEC and UFF sEVs (Figure 5). EVs of plasma membrane origin are often characterised by the protein marker ANXA2. Interestingly, ANXA2 was enriched in SEC sEVs compared to UFF sEVs (Table S3). Overall, proteomic analyses reveals that SEC and UFF enrich for different subsets of sEVs.

The presence of glycoproteins on sEVs reflects post‐translational modifications that may influence retention characteristics during sEV isolation (Yang et al. 2017; Williams et al. 2018). Consistent with this, differential abundance analysis identified various glycoproteins (Figure S9). Of the glycoproteins detected by mass spectrometry, 45 and 57 proteins were significantly enriched in SEC and UFF sEVs, respectively. The majority of identified glycoproteins were annotated as N‐glycosylated, consistent with the predominance of secreted and membrane‐associated proteins in sEVs (Zhang et al. 2021). A small subset exhibited O‐linked glycosylation, primarily associated with mucin‐like domains or extracellular domains (Wandall et al. 2021) (Figure S9).

Furthermore, gene ontology (GO) analysis revealed an enrichment of proteins associated with collagen‐containing extracellular matrix in SEC sEVs, and blood microparticles in UFF sEVs (Figure S10A,B). For biological processes, proteins involved in humoral immune response and wound healing were enriched in SEC and UFF sEVs, respectively (Figure S10A,B). Whereas proteins with molecular functions in extracellular matrix structural constituents and serine‐type endopeptidase activity were enriched in SEC and UFF sEVs, respectively (Figure S10A,B).

### 3.5 SEC Is Cost‐effective With the Capacity for Scalability, Whilst UFF Is Automated and Time‐Efficient

Due to its gentle isolation principle, scalability and economic feasibility, SEC is widely accepted as a very effective method for plasma EV isolation (Lobb et al. 2015; Clos‐Sansalvador et al. 2022; Dilsiz 2024). However, the process is performed manually (Table S4). Hence the appeal of automated EV isolation systems, such as UFF (Chen et al. 2021). This has caused a shift in the current market towards the development and use of these types of semi‐automated platforms (Kapoor et al. 2024). The SEC columns vary greatly in their performance in regards to flow rates, so the time taken to complete a single isolation of sEVs from plasma, fluctuates (Table S4). In terms of cost efficacy and scalability, SEC would be the better alternative, with a 2‐fold difference in costs and unlimited scalability potential (Table S4).

## 4 Discussion

The implementation of EVs in clinical and commercial settings requires an EV isolation method that is both time‐ and cost‐effective, and capable of producing high particle yields with the desired genetic cargo. However, isolating sEVs from plasma presents several challenges, elevated lipid and protein content, and the presence of platelets (Stranska et al. 2018).

Plasma viscosity increases with the presence of plasma proteins, such as fibrinogen, immunoglobulins, chylomicrons and VLDLs (Nader et al. 2019; Seplowitz et al. 1981). With the 70 nm SEC qEV column (IZON), elevated plasma viscosity may reduce flow rates, impeding the efficient movement of sEVs through the porous matrix. High protein concentrations may cause matrix saturation, potentially compromising separation, resolution and recovery rates, resulting in co‐elution and thereby affecting the EV output (Nader et al. 2019; Benayas et al. 2023). In UFF systems, increased plasma viscosity may exacerbate filtration resistance, prolonging processing times and potentially preventing complete filtration of smaller particles. Additionally, high protein concentrations in UFF systems may trigger membrane clogging, diminish overall filtration efficiency and complicate downstream analysis. The presence of various EV populations, such as Platelet‐derived EVs (PDEVs), can also interfere with the functional analyses of desired sEVs. PDEVs constitute the majority of EVs detected in the plasma and may therefore be co‐isolated during plasma‐based EV enrichment processes (Kumar et al. 2024). As PDEVs overlap in the size and density with other sEV populations, their co‐isolation may further influence particle yield and molecular composition (Vaiaki and Falasca 2024).

For the utilisation of EVs as drug delivery transporters, therapeutic tools and biomarker carriers, the miRNA content and proteomic profiles of EVs are arguably the leading factor in considering the most optimal EV isolation method. Within plasma, substantial quantities of miRNAs are associated with free proteins, consequently, the accurate detection of sEV‐derived miRNAs is important (Xu et al. 2022). The miRNA sequencing revealed isolation method‐dependent differences in miRNA detection between SEC‐sEVs and UFF‐sEVs, likely reflecting inherent variations in isolation efficiency and vesicle recovery (Srinivasan et al. 2019; Tang et al. 2017; Godoy et al. 2019). A comparatively lower number of miRNAs exclusively detected in UFF sEVs may indicate particle loss or degradation. Therefore, these method‐dependent variations may impact downstream biomarker analysis (Mercadal et al. 2020; Buschmann et al. 2018).

The EV proteome largely determines the fate of EVs in biological processes (Lima et al. 2021; Jimenez et al. 2019; Lischnig et al. 2022); hence the presence and/or absence of specific proteins can heavily impact research and/or clinical outcomes. It is essential to ensure that the particles isolated carry EV‐associated proteins so that any effect observed is a reliable and accurate representation of EVs and not of non‐EV materials (Buzás et al. 2018). Canonical EV tetraspanins and integrins have been reported as potential biomarkers of diseases, ranging from cancer (Rydland et al. 2023; Hoshino et al. 2015; Kawakami et al. 2015; Hurwitz and Meckes 2019) to kidney disease (Hogan et al. 2014) to sepsis (Im et al. 2021; Mackraj et al. 2024; Kawamoto et al. 2019). Importantly, EV‐associated tetraspanins and integrins are heavily involved in the crosstalk between EVs and the extracellular matrix, due to their ligand binding capacities, playing major roles in EV‐mediated uptake, homing, organotropism and signal transduction (Hoshino et al. 2015; Singh et al. 2016; Park et al. 2019; Nigri et al. 2022; Oh et al. 2024; Dai et al. 2022; Park et al. 2025; Isogai et al. 2025). The enrichment of tetraspanins CD9 and CD81 in SEC sEVs, and integrins ITGA1, ITGA2, ITGB1 and LAMP2 in UFF sEVs, suggests that both methods isolate different sEV subpopulations.

Isolation of sEVs results in a mixture of particles that originate from endosomes and the plasma membrane (Jeppesen et al. 2019; Bano et al. 2021). Separation and subsequent analyses of sEVs by size, density, immunoaffinity and biogenesis have shown significant differences in the abundance of tetraspanins and integrins (Jimenez et al. 2019; Kowal et al. 2016; Fordjour et al. 2022; Mathieu et al. 2021). Tetraspanins and integrins are not necessarily present on the same particles, as it has been reported that EVs derived from the same source can be enriched in either tetraspanins or integrins or they can be colocalised together (Serru et al. 1999; Kaur et al. 2022). Several studies have proposed that CD9 and CD81 are localised within the plasma membrane, whereas CD63 is localised within late endosomes, suggesting that their presence is detected in different sEV subtypes (Fordjour et al. 2022, Mathieu et al. 2021). Similarly, various integrins including ITGA1, ITGA2, ITGB1, LAMP2 and others, have been reported to be associated with different sEV subtypes (Isogai et al. 2025; Surman et al. 2021; Meldolesi 2018; Altei et al. 2020). Particles of plasma membrane origin are generally secreted in higher concentrations compared to particles of endosomal origin (Mathieu et al. 2021), which may explain the overall low abundance of CD63 in both SEC and UFF sEV isolations. EVs enriched in tetraspanins have different effects on recipient cells compared to those enriched in integrins (Kaur et al. 2022). Similarly, particles derived from endosomes and the plasma membrane exert different functional effects (Xu et al. 2024). Although EV surface proteins are critical for binding to target cells, their binding capacities can be masked by the presence of outer proteins, reducing therapeutic specificity.

Plasma has a complex composition due to the coexistence of other molecules. Due to the high abundance of non‐vesicular, soluble proteins in blood (Lucien et al. 2023; Nieuwland and Siljander 2024), the majority of total protein measured is likely attributable to non‐EV protein. The biomolecular corona describes an array of proteins that decorate the surface of EVs, acquired either intracellularly or upon secretion into the extracellular space (Tóth et al. 2021; Esmaeili et al. 2025; Liam‐Or et al. 2024). The corona is an emerging, sometimes controversially discussed topic, as some proteins that were once considered ‘contaminants’ are now increasingly recognised as potential constituents of EV cargo (Lucien et al. 2023; Wolf et al. 2022; Singh et al. 2023; Yerneni et al. 2022). Various components are classified as corona proteins, including albumin, apolipoproteins, immunoglobulins, and complement and fibrinogen proteins. However, these proteins can be present either in their soluble, non‐EV bound form, or associated with the EV corona, thereby complicating the distinction between these two forms. The importance of the presence or absence of corona proteins is highly dependent on the context in which the EVs isolated are used. For biomarker discovery and detection, a high abundance of blood proteins may mask low abundance EV proteins, decreasing sensitive and specific detection of target proteins. For EV‐based therapies, the presence of corona proteins must enable high biocompatibility, effectively induce targeted cell binding or uptake, evade immune responses and prolong circulation to ensure appropriate function. The proteins responsible for these functions are purely dependent on the EV source, the intended target and the application. This is further complicated as different biological samples vary markedly in biochemical composition. It has been previously demonstrated that UFF has notable efficiency in purifying EVs from low volumes (ten microlitres to ten millilitres) of various biological fluids, including tears (Hu et al. 2022; Hu et al. 2023), cerebrospinal fluid (Li et al. 2021), and urine (Zhu et al. 2022; Mao et al. 2025; Jiang et al. 2024). Thus, there is a need to standardise EV isolation workflows according to the properties of the sample, as viscosity, protein content and matrix complexity influence co‐elution of non‐EV proteins (Martins et al. 2023; Koster et al. 2021; Mangolini et al. 2024). Adapting pre‐processing and isolation parameters to the characteristics of the starting material may reduce the co‐isolation of soluble proteins and may enhance the interpretability of downstream genomic and proteomic data.

Glycosylation plays a crucial role in the biosynthesis, cargo sorting, uptake, stability, and tissue‐specific targeting of sEVs (Williams et al. 2018; Zhou et al. 2024). These surface glycans are crucial for cell recognition and immune regulation, while also serving as valuable biomarkers for detecting diseases such as cancer, where abnormal glycosylation patterns often occur (Wu and Gao 2023; Lin et al. 2020). Extensive N‐glycosylation of EV transmembrane and secretory proteins may modulate the hydrodynamic radius and surface charge density of vesicles. This modification interferes with the separation matrix, ultimately affecting recovery rates and the depth of downstream proteomics analysis (Yang et al. 2017; Williams et al. 2018). O‐linked glycans typically aggregate on mucin‐like proteins, forming dense surface structures that increase steric hindrance, alter the effective hydrodynamic diameter, and result in underrepresentation in mass spectrometry (Bechtella et al. 2024; Du et al. 2020). N‐glycosylation is crucial for the interaction between sEVs and receptor cells, hence enzymatic removal of N‐glycans has been shown to reduce EV uptake and attenuate downstream signalling pathways, such as angiogenesis (Rocamora et al. 2023; Clos‐Sansalvador et al. 2022). In parallel, O‐glycosylation affects immune clearance and biodistribution dynamics (Rocamora et al. 2023; Lundahl et al. 2021). Alterations in N‐ and O‐ linked glycan profiles, particularly those produced by non‐cancerous cells, have therefore emerged as effective biomarkers for cancer diagnosis, prognosis and treatment monitoring (Lan et al. 2016).

As the most abundant circulatory protein, the co‐isolation of albumin in EV preparations is inevitable (Askeland et al. 2020). Albumin plays a major role in the transportation of molecular cargo in the blood (Wu et al. 2024; van de Wouw and Joles 2022). It has been previously shown that EVs enriched in albumin attract additional adsorption of albumin to the EV surface and less adsorption of immunoglobulins and complement factors (Liam‐Or et al. 2024). This supports the mass spectrometry findings of the enrichment of albumin and decreased abundance of immunoglobulins and complement proteins in UFF sEVs. Numerous albumin depletion kits are commercially available, as studies have demonstrated that albumin depletion results in the enrichment of low abundance proteins (Zhang et al. 2023; Liu et al. 2014). However, the removal of albumin may also result in the removal of other target proteins (Zhang et al. 2023). In regards to the effect of albumin on EV and cellular function, previous literature have reported the advantages of albumin in enhancing EV function (Liam‐Or et al. 2024; Liang et al. 2022), by extending circulation time (Liang et al. 2022) and increasing uptake by recipient cells in *in vivo* murine models (Liam‐Or et al. 2024). Although SEC effectively reduces albumin, which has also been shown by others (Stranska et al. 2018; Baranyai et al. 2015), it is not completely removed.

The overlap in size and density range results in the co‐isolation of apolipoproteins with EVs, hindering NTA from distinguishing EVs from NVEPs, thus skewing particle quantification (Yuana et al. 2014). Despite the potential interference of NVEPs, no significant difference in particle‐to‐lipid ratios was observed between SEC sEVs and UFF sEVs. Relative comparisons based on particle number are reliable, and are unlikely to be substantially influenced by NVEP contamination. Many researchers desire the elimination of apolipoproteins for the purpose of biomarker discovery, proteomic and lipidomic analyses and inflammation studies (Yuana et al. 2014; Zhang et al. 2020; Merij et al. 2024). Apolipoproteins are dysopsonins, prolonging circulation time and avoiding phagocytic clearance by monocytes, increasing interactions with target tissues (Dietz et al. 2023). Here, we found an enrichment of high‐density lipoproteins ApoA1, ApoA2, ApoD and ApoH in UFF sEVs. ApoA1 and ApoD have been previously reported in the EV corona (Tóth et al. 2021; Dietz et al. 2023; Wolf et al. 2022; Karimi et al. 2018; Cheng et al. 2021) with the ability to enhance EV uptake for the delivery of EV cargo and consequent elicitation of cellular changes (Liang et al. 2018; Pascua‐Maestro et al. 2018). The colocalization of ApoA1 and haptoglobin have been previously reported (Tóth et al. 2021), which may explain the enrichment of both molecules in UFF sEVs. Other apolipoproteins such as ApoB, ApoC3 and ApoE have also been reported as universal corona proteins (Tóth et al. 2021), which are all of comparable abundance between SEC sEVs and UFF sEVs. Interestingly, high‐density lipoproteins have a similar range of density to EVs, however are much smaller in size in comparison (Tóth et al. 2021; Karimi et al. 2018). It is possible that the presence of high‐density lipoproteins in UFF sEVs reduced the median particle size, resulting in smaller particle diameters.

The presence of immunoglobulins and fibrinogen proteins in the EV protein corona (Tóth et al. 2021; Dietz et al. 2023; Shtam et al. 2018) has been demonstrated in previous studies, as treatment of EVs with NaCl and trypsin resulted in the disruption of immunoglobulins and fibrinogen with the EV surface (Tóth et al. 2021; Shtam et al. 2018). The presence of various immunoglobulins and fibrinogen proteins may attribute to the larger particle populations isolated by SEC, as the EV corona increases the hydrodynamic size of EV particles (Tóth et al. 2021; Shtam et al. 2018). Immunoglobulins and fibrinogen proteins have been previously reported as potential plasma EV biomarkers for cancer (Yang et al. 2023; Couto et al. 2022; Capello et al. 2019; Hoshino et al. 2020; Kuang et al. 2019; Sun et al. 2021), cardiovascular disease (Moreira‐Costa et al. 2021) and liver disease (Cho et al. 2017). However, their exact contribution to EV‐mediated processes has not been thoroughly explored. Immunoglobulins bind Fc receptors, typically expressed on the surface of immune cells, to induce various cellular effects (Pincetic et al. 2014; Mancardi and Daëron. 2014; Daëron 2014). One study reported that the protein corona of colorectal cancer‐derived EVs largely consisted of immunoglobulins. However, blocking Fc receptors on recipient monocytes, had no effect on the uptake of the colorectal cancer‐derived EVs, suggesting that immunoglobulins had no significant role in EV uptake (Dietz et al. 2023). Further research is required to determine the importance of immunoglobulins and fibrinogens in downstream EV applications.

## 5 Conclusion

For the commercial and clinical use of sEVs, there is a fine balance between prioritising isolation time consumption, cost efficacy, scalability, and sEV quantity and quality. Due to the wealth of genetic information contained within sEVs and its functional abilities, the proteomic cargo is arguably the most valuable parameter to consider when choosing an isolation method. Overall, various intravesicular and corona‐bound proteins were detected in both SEC and UFF sEV preparations, so there appears to be no specific retention and/or elimination of certain protein groups. Hence, it is encouraged that researchers choose the best isolation method for their applications, whether that is the use of plasma sEVs as biomarkers or as therapeutic agents in the treatments of disease.

## Author Contributions

**Yue Su**: conceptualization, investigation, writing – original draft, writing – review and editing. **Kekoolani S. Visan**: conceptualization, investigation, funding acquisition, supervision, writing – original draft, writing – review and editing. **Sarah Voss**: investigation, writing – original draft, writing – review and editing. **Marta Prieto–Vila**: investigation, supervision, writing – review and editing. **Juntaro Matsuzaki**: conceptualization, investigation, supervision, writing – review and editing. **Jason Ying Kuen Chan**: conceptualization, investigation, supervision, resources, writing – review and editing. **Judy Wai Ping Yam**: conceptualization, investigation, supervision, resources, writing – review and editing. **Andreas Möller**: conceptualization, investigation, funding acquisition, supervision, resources, writing – original draft, writing – review and editing.

## Conflicts of Interest

The authors declare no conflicts of interest.

## Supporting information




**Supplementary Figure 1. UC isolates particles within the sEV size range. (A)** Graphical depiction of the principle of UC‐based isolation of sEVs. **(B)** Size distribution, **(C)** particle concentration, **(D)** median particle diameter (nm), percentage of particles **(E)** smaller than 100 nm, **(F)** between 100 and 200 nm in diameter and **(G)** larger than 200 nm, were assessed by nanoparticle tracking analysis. **(H)** Protein concentration measured by Bradford Assay. **(I)** Number of particles per microgram of protein. Data are presented as *n* = 8±S.E.M. UC: Ultracentrifugation.

**Supplementary Figure 2. SEC isolates a higher percentage of large EVs compared to UFF**. Size distribution of **(A)** SEC and **(B)** UFF sEVs, and percentage of particles **(C)** smaller than 100 nm, **(D)** between 100 and 200 nm in diameter and **(E)** larger than 200 nm, were assessed by nanoparticle tracking analysis. Representative transmission electron microscopy images show the larger background of particles isolated by **(F)** SEC and **(G)** UFF. The red arrow points to sEV. Size bar is 500 nm. Data are presented as *n* = 10±SEM. **p <0.01. Statistical analyses were performed using paired *t*‐test, Wilcoxon test. SEC: Size Exclusion Chromatography; UFF: Ultrafast Filtration.

**Supplementary Figure 3. Vast differences in particle and protein amounts between sEVs isolated by SEC and UFF result in varying detection of EV and non‐EV‐associated proteins. (A)** Western blot analyses of EV protein markers HSP70, TSG101, Flotillin‐1 and CD81 in plasma (P) and EVs isolated by SEC and UFF. The serum protein Albumin was used to assess the presence of secreted proteins typically recovered with blood‐derived EVs. Equal particle numbers (1.0 × 10^10^ particles) and equal protein amounts (5.0 µg) of EVs were loaded. The corresponding protein and particle amounts are shown. **(B)** Capillary‐based western blot analyses of EV protein markers Alix, HSP70, Flotillin‐1, Syntenin‐1 and CD81 in plasma (P) and EVs isolated by SEC and UFF. The exocytosis protein Annexin A1 and serum proteins C3, Albumin, ApoA1 and ApoE, were used to assess the presence of large EVs and secreted proteins typically recovered with blood‐derived EVs. Equal particle numbers (1.0 × 10^10^ particles) were loaded. The corresponding protein amounts are shown. **(C)** Proteinase K digestion assay on EVs isolated by SEC and UFF, treated 5 min with increasing concentrations of Proteinase K, sEVs untreated with proteinase K and plasma (P) as control groups. Expression of immunoglobulin IgG4, apolipoprotein ApoE, and surface EV marker CD81 was assessed. Equal protein amounts (5.0 µg) of EVs were loaded. SEC: Size Exclusion Chromatography; UFF: Ultrafast Filtration.

**Supplementary Figure 4. SEC Isolates a Higher Amount of miRNA Compared to UFF. (A)** Correlation of miRNA expression levels between individual samples. Scatter plots show the correlation of miRNA expression between SEC and UFF pairs. The Pearson correlation coefficient (R) and its 95% confidence interval are indicated for each comparison. Data are presented as *n* = 5±SEM. ***p <0.005. Statistical analyses were performed using paired t‐test. SEC: Size Exclusion Chromatography; UFF: Ultrafast Filtration.

**Supplementary Figure 5. SEC isolates a higher number of CD9^+^ particles compared to UFF**. ELISA measuring the number of **(A)** CD9^+^, **(B)** CD81^+^ and **(C)** CD63^+^ particles. Data are presented as *n* = 8±SEM. **p <0.01. Statistical analyses were performed using paired t‐test, Wilcoxon test. SEC: Size Exclusion Chromatography; UFF: Ultrafast Filtration.

**Supplementary Figure 6. UFF isolates a higher abundance of Albumin and apolipoproteins from plasma compared to SEC**. Heat map of major components of non‐EV co‐isolated proteins detected in SEC and UFF sEVs, as determined by mass spectrometry. The colour key denotes the row z‐score. Data are presented as *n* = 8. SEC: Size Exclusion Chromatography; UFF: Ultrafast Filtration.

**Supplementary Figure 7. SEC and UFF isolate particles enriched in different secreted proteins**. ELISA measuring the abundance of **(A)** C3 and **(B)** MAC2BP abundance. **(C)** Heat map of secreted proteins recovered with EVs, as determined by mass spectrometry. The colour key denotes the row z‐score. Data are presented as *n* = 8±SEM. *p <0.05. SEC: Size Exclusion Chromatography; UFF: Ultrafast Filtration.

**Supplementary Figure 8. SEC isolates a higher abundance of immunoglobulins**. Heat map of immunoglobulins, as determined by mass spectrometry. The colour key denotes the row z‐score. Data are presented as *n* = 8. SEC: Size Exclusion Chromatography; UFF: Ultrafast Filtration.

**Supplementary Figure 9. A graphical summary of glycoproteins significantly enriched in sEVs isolated by SEC and UFF, identified by mass spectrometry. (A)** SEC and **(B)** UFF sEVs are denoted by the blue and purple outlined box, respectively. Glycoproteins of SEC and UFF sEVs are denoted by the black outlined box, glycoproteins that undergo both N‐glycosylation and O‐glycosylation are denoted by the red outlined box.

**Supplementary Figure 10. Gene Ontology analysis of proteins enriched in (A) SEC and (B) UFF sEVs**. BP: biological process; CC: cellular component; MF: molecular function; SEC Size Exclusion Chromatography; UFF: Ultrafast Filtration.

**Supplementary Table 1. Particle and protein concentrations of sEVs isolated by SEC and UFF**.

**Supplementary Table 2. List of microRNAs consistently detected in all five samples within the SEC and UFF groups by next‐generation sequencing**.

**Supplementary Table 3**. List of proteins identified by mass spectrometry.

**Supplementary Table 4. Characteristics of sEVs isolation methods**. To facilitate comparison of the isolation methods described below, a relative scoring system was implemented for each parameter: + (low), ++ (intermediate), +++ (high), or ++++ (very high). SEC: Size Exclusion Chromatography; UFF: Ultrafast Filtration.

## Data Availability

The data that supports the findings of this study are available in the supplementary material of this article
