# Supplementary figures and images for: Impact of Isolation Techniques on the Content of Small Extracellular Vesicles
Source: J Extracell Vesicles. 2026 May 28;15(6):e70290. doi: 10.1002/jev2.70290 (PMC13239987; doi:10.1002/jev2.70290)

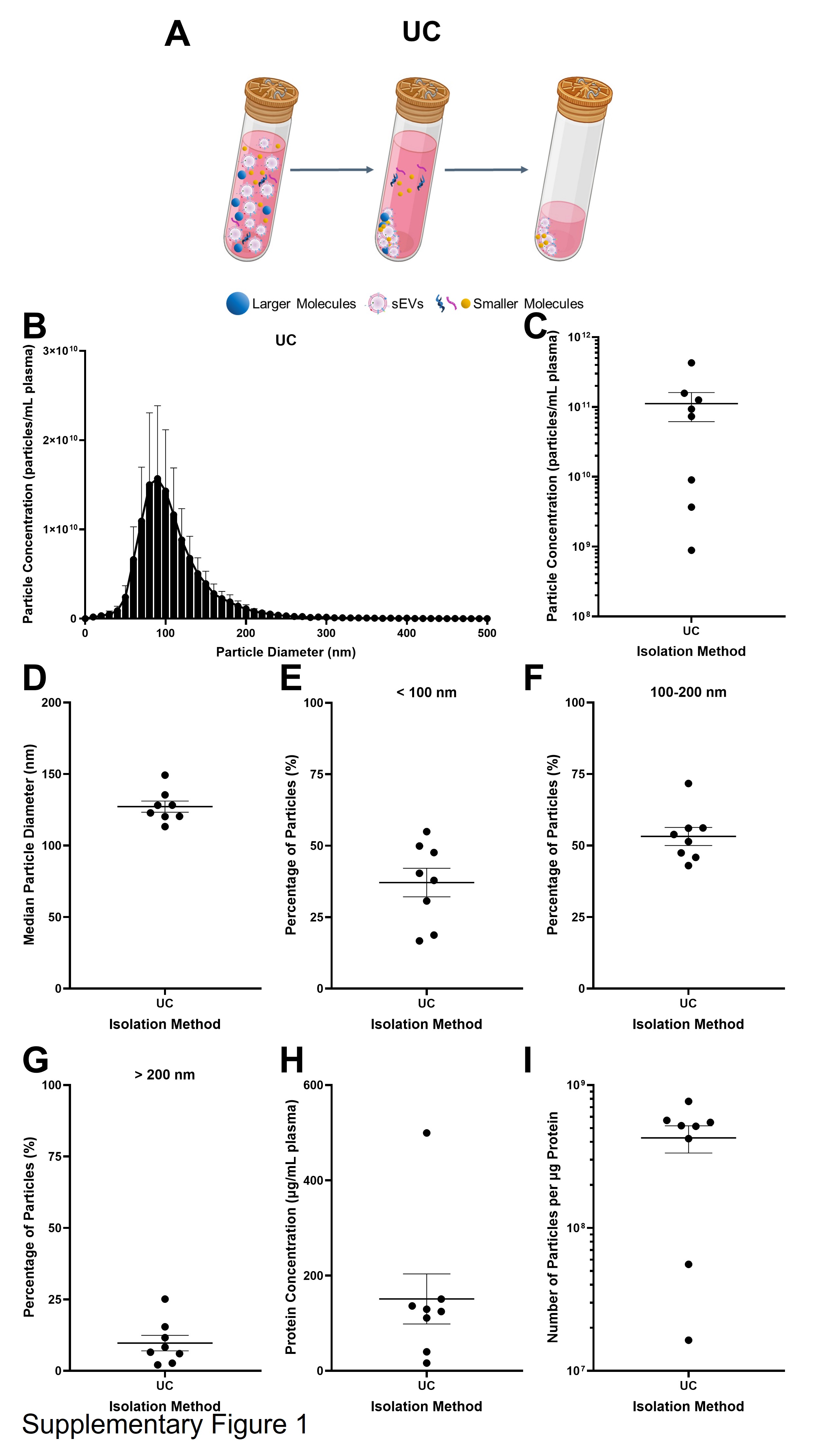

Supplement: Supplementary file 1 — Supplementary Figure 1. UC isolates particles within the sEV size range. (A) Graphical depiction of the principle of UC‐based isolation of sEVs. (B) Size distribution, (C) particle concentration, (D) median particle diameter (nm), percentage of particles (E) smaller than 100 nm, (F) between 100 and 200 nm in diameter and (G) larger than 200 nm, were assessed by nanoparticle tracking analysis. (H) Protein concentration measured by Bradford Assay. (I) Number of particles per microgram of protein. Data are presented as n = 8±S.E.M. UC: Ultracentrifugation. [file JEV2-15-e70290-s007.jpg]

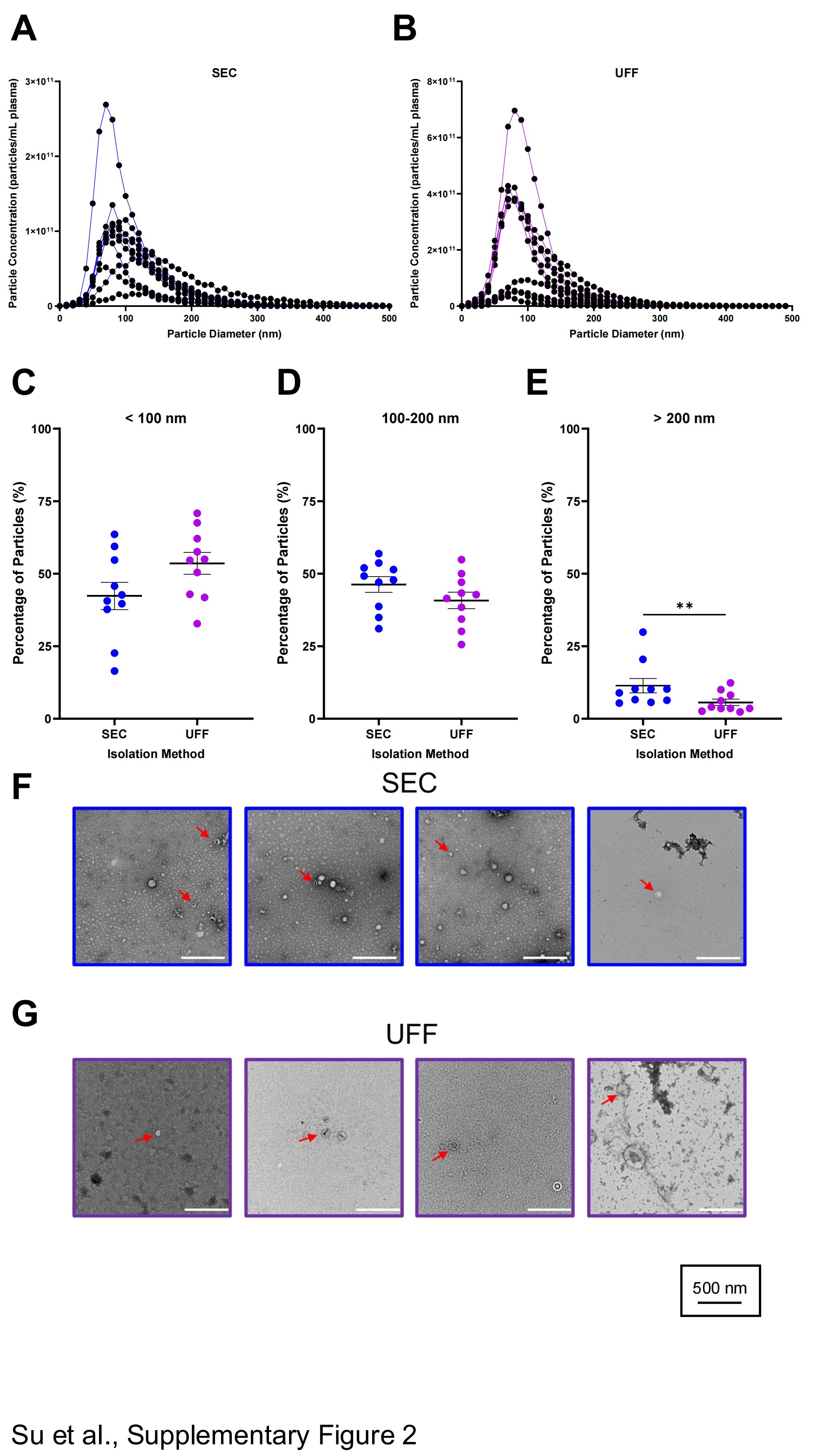

Supplement: Supplementary file 2 — Supplementary Figure 2. SEC isolates a higher percentage of large EVs compared to UFF. Size distribution of (A) SEC and (B) UFF sEVs, and percentage of particles (C) smaller than 100 nm, (D) between 100 and 200 nm in diameter and (E) larger than 200 nm, were assessed by nanoparticle tracking analysis. Representative transmission electron microscopy images show the larger background of particles isolated by (F) SEC and (G) UFF. The red arrow points to sEV. Size bar is 500 nm. Data are presented as n = 10±SEM. **p <0.01. Statistical analyses were performed using paired t‐test, Wilcoxon test. SEC: Size Exclusion Chromatography; UFF: Ultrafast Filtration. [file JEV2-15-e70290-s009.jpg]

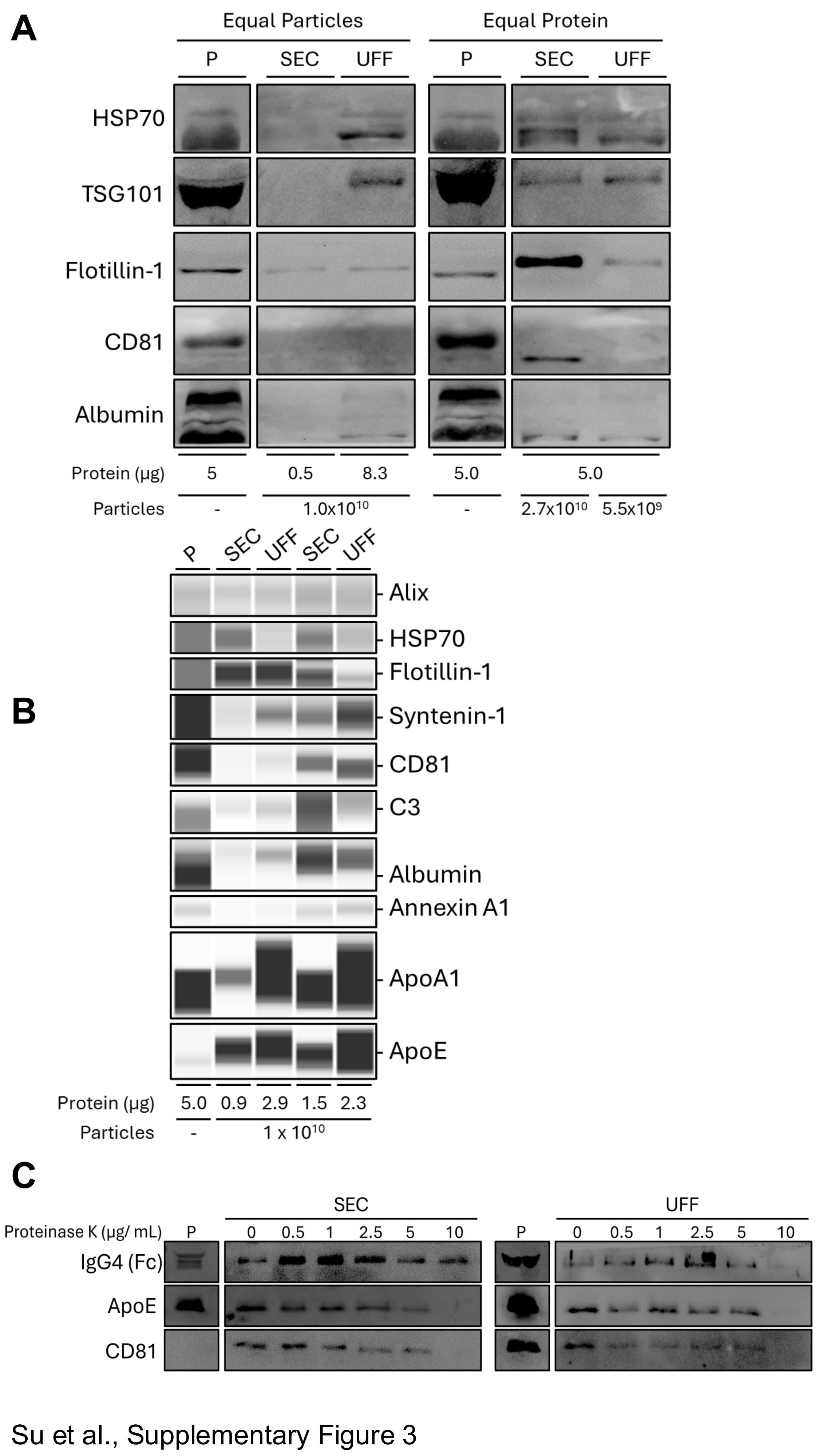

Supplement: Supplementary file 3 — Supplementary Figure 3. Vast differences in particle and protein amounts between sEVs isolated by SEC and UFF result in varying detection of EV and non‐EV‐associated proteins. (A) Western blot analyses of EV protein markers HSP70, TSG101, Flotillin‐1 and CD81 in plasma (P) and EVs isolated by SEC and UFF. The serum protein Albumin was used to assess the presence of secreted proteins typically recovered with blood‐derived EVs. Equal particle numbers (1.0 × 10^10^ particles) and equal protein amounts (5.0 µg) of EVs were loaded. The corresponding protein and particle amounts are shown. (B) Capillary‐based western blot analyses of EV protein markers Alix, HSP70, Flotillin‐1, Syntenin‐1 and CD81 in plasma (P) and EVs isolated by SEC and UFF. The exocytosis protein Annexin A1 and serum proteins C3, Albumin, ApoA1 and ApoE, were used to assess the presence of large EVs and secreted proteins typically recovered with blood‐derived EVs. Equal particle numbers (1.0 × 10^10^ particles) were loaded. The corresponding protein amounts are shown. (C) Proteinase K digestion assay on EVs isolated by SEC and UFF, treated 5 min with increasing concentrations of Proteinase K, sEVs untreated with proteinase K and plasma (P) as control groups. Expression of immunoglobulin IgG4, apolipoprotein ApoE, and surface EV marker CD81 was assessed. Equal protein amounts (5.0 µg) of EVs were loaded. SEC: Size Exclusion Chromatography; UFF: Ultrafast Filtration. [file JEV2-15-e70290-s006.jpg]

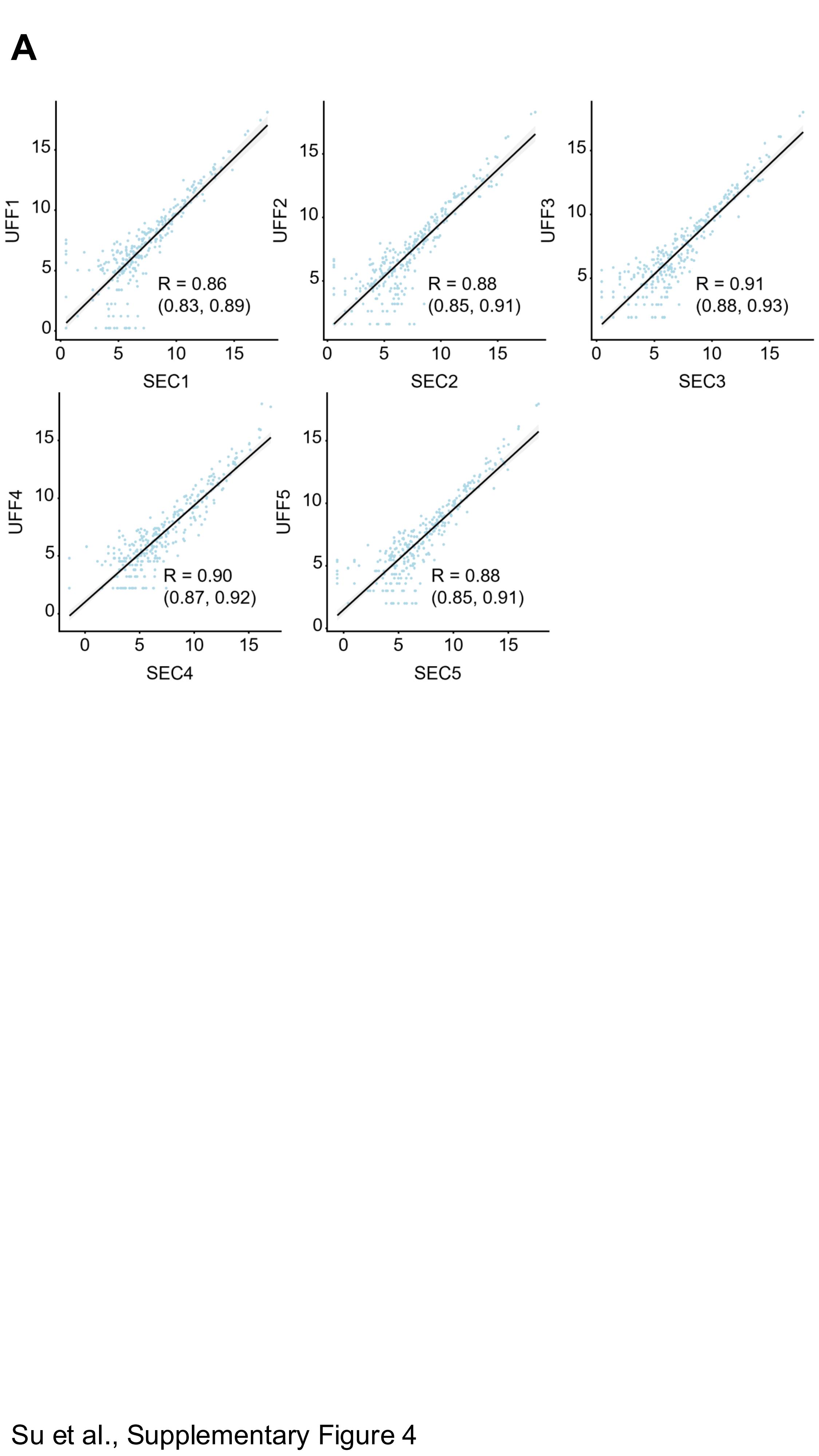

Supplement: Supplementary file 4 — Supplementary Figure 4. SEC Isolates a Higher Amount of miRNA Compared to UFF. (A) Correlation of miRNA expression levels between individual samples. Scatter plots show the correlation of miRNA expression between SEC and UFF pairs. The Pearson correlation coefficient (R) and its 95% confidence interval are indicated for each comparison. Data are presented as n = 5±SEM. ***p <0.005. Statistical analyses were performed using paired t‐test. SEC: Size Exclusion Chromatography; UFF: Ultrafast Filtration. [file JEV2-15-e70290-s011.jpg]

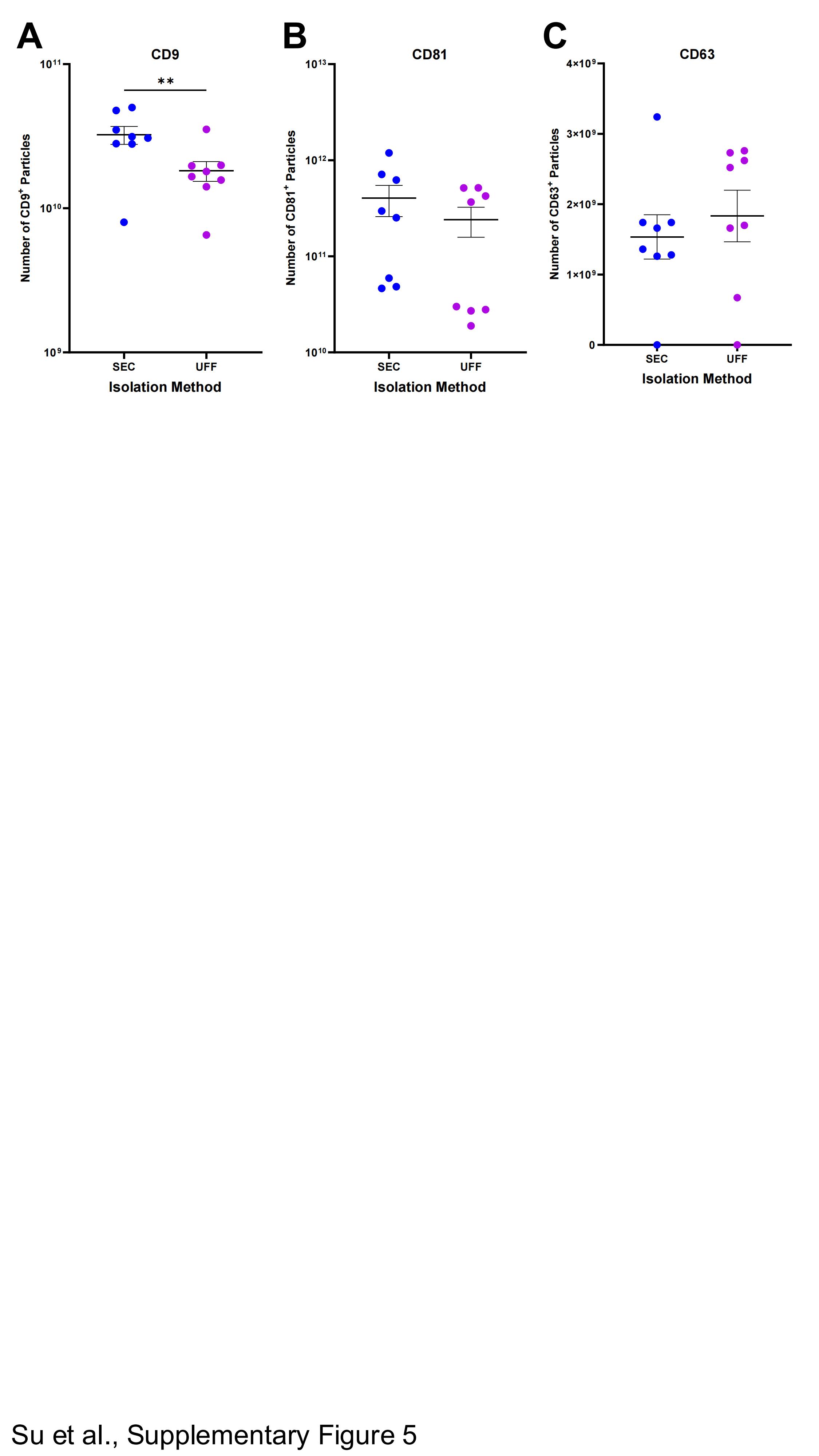

Supplement: Supplementary file 5 — Supplementary Figure 5. SEC isolates a higher number of CD9+ particles compared to UFF. ELISA measuring the number of (A) CD9+, (B) CD81+ and (C) CD63+ particles. Data are presented as n = 8±SEM. **p <0.01. Statistical analyses were performed using paired t‐test, Wilcoxon test. SEC: Size Exclusion Chromatography; UFF: Ultrafast Filtration. [file JEV2-15-e70290-s001.jpg]

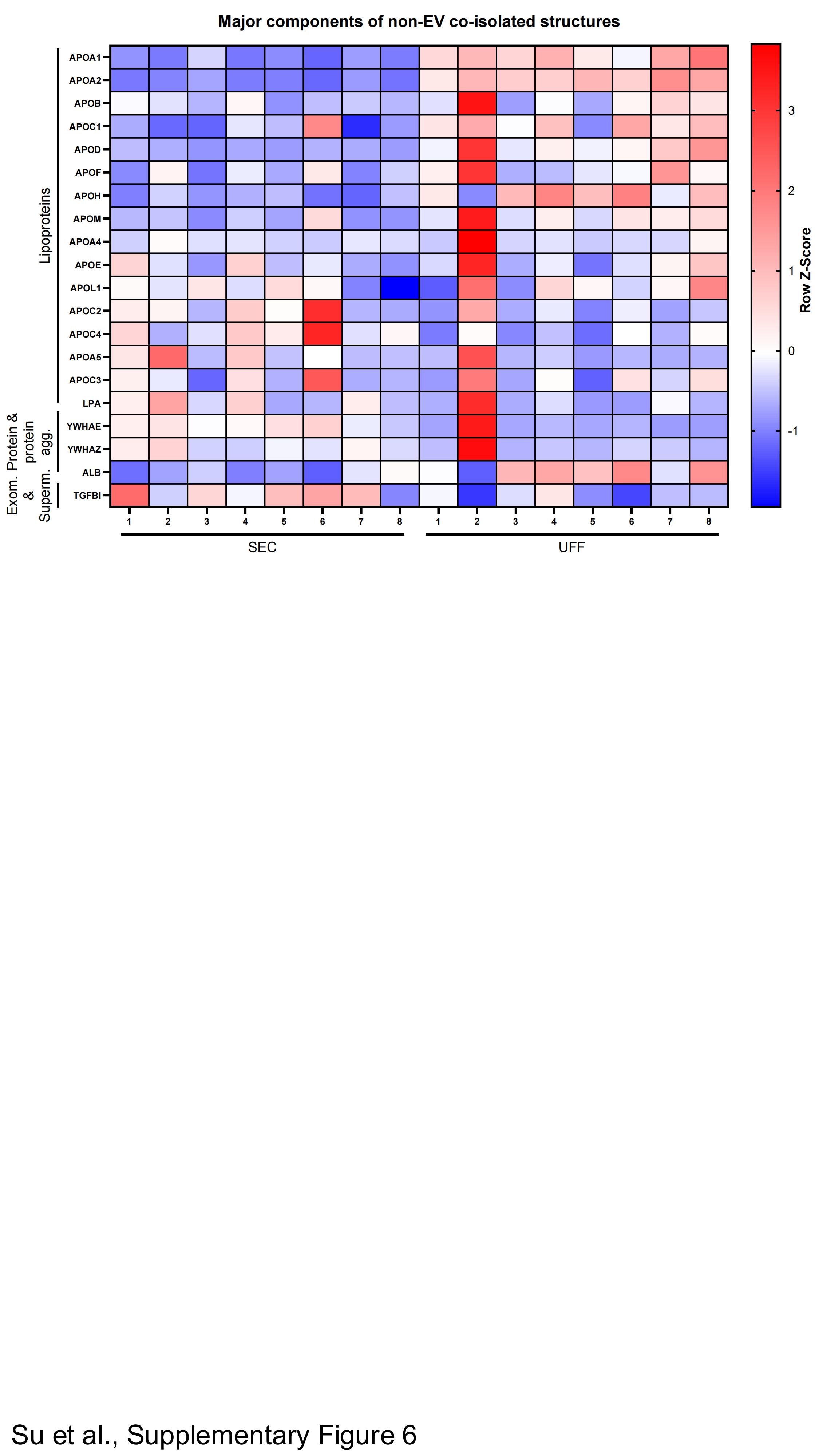

Supplement: Supplementary file 6 — Supplementary Figure 6. UFF isolates a higher abundance of Albumin and apolipoproteins from plasma compared to SEC. Heat map of major components of non‐EV co‐isolated proteins detected in SEC and UFF sEVs, as determined by mass spectrometry. The colour key denotes the row z‐score. Data are presented as n = 8. SEC: Size Exclusion Chromatography; UFF: Ultrafast Filtration. [file JEV2-15-e70290-s002.jpg]

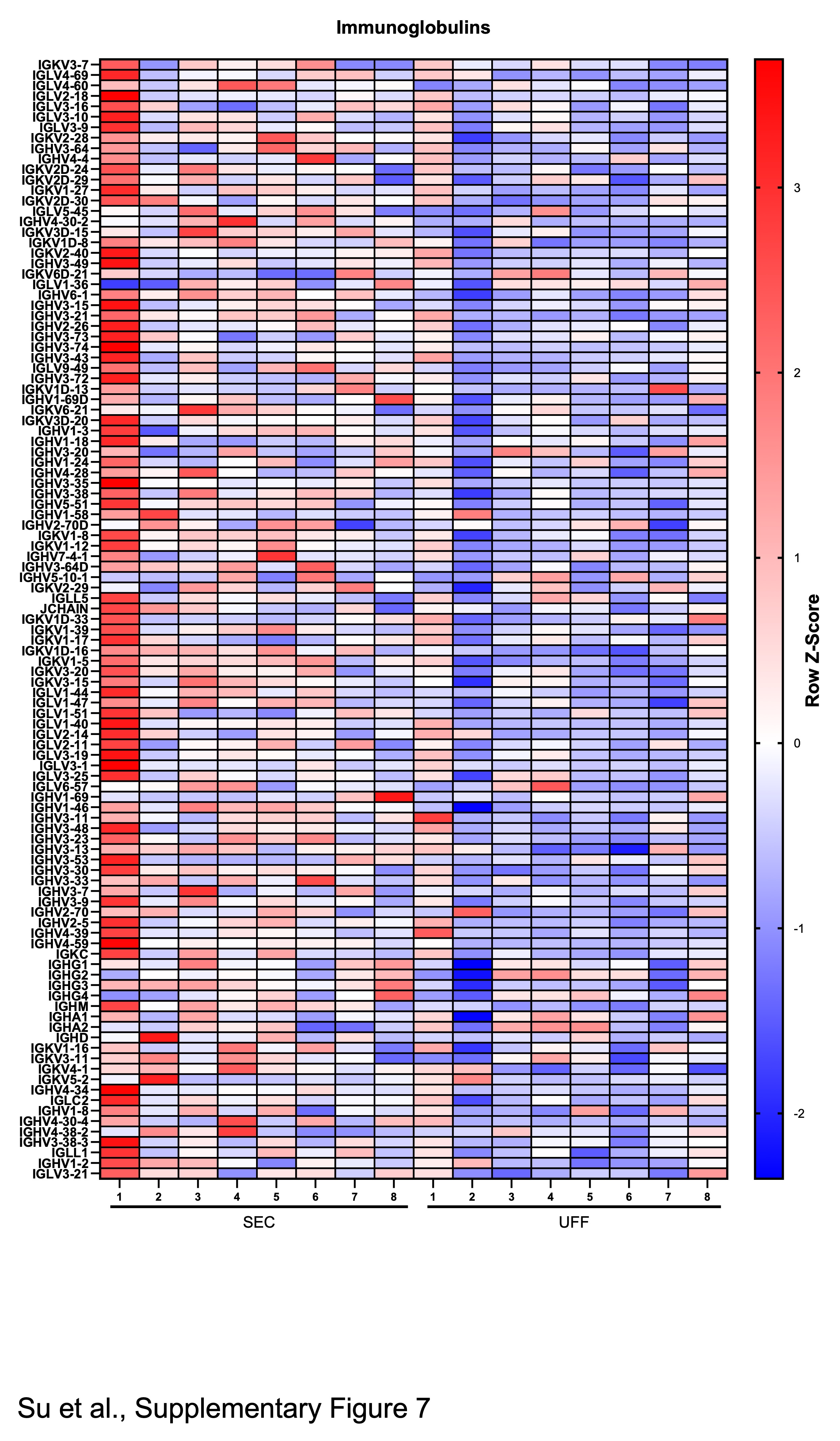

Supplement: Supplementary file 7 — Supplementary Figure 7. SEC and UFF isolate particles enriched in different secreted proteins. ELISA measuring the abundance of (A) C3 and (B) MAC2BP abundance. (C) Heat map of secreted proteins recovered with EVs, as determined by mass spectrometry. The colour key denotes the row z‐score. Data are presented as n = 8±SEM. *p <0.05. SEC: Size Exclusion Chromatography; UFF: Ultrafast Filtration. [file JEV2-15-e70290-s012.jpg]

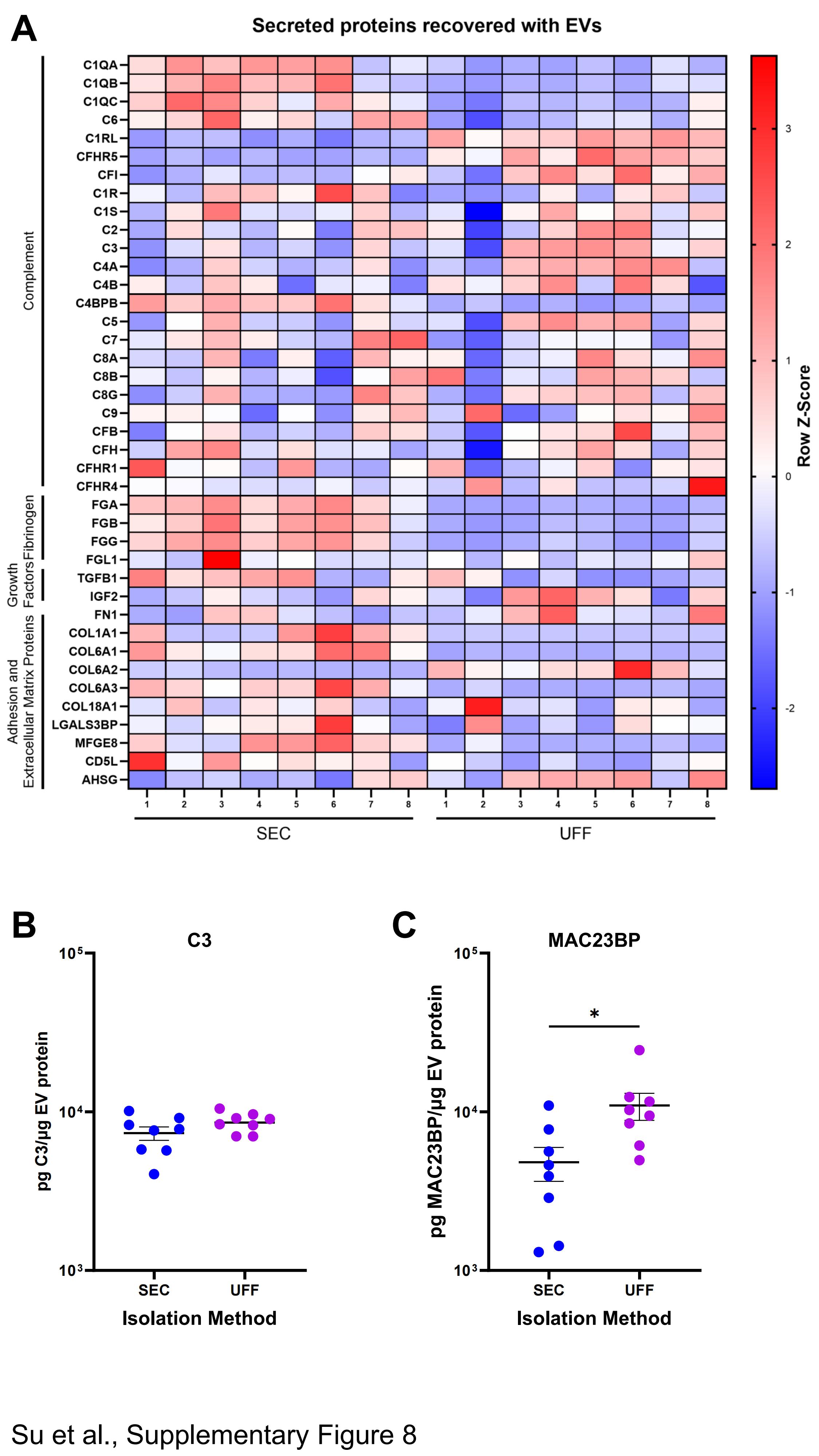

Supplement: Supplementary file 8 — Supplementary Figure 8. SEC isolates a higher abundance of immunoglobulins. Heat map of immunoglobulins, as determined by mass spectrometry. The colour key denotes the row z‐score. Data are presented as n = 8. SEC: Size Exclusion Chromatography; UFF: Ultrafast Filtration. [file JEV2-15-e70290-s003.jpg]

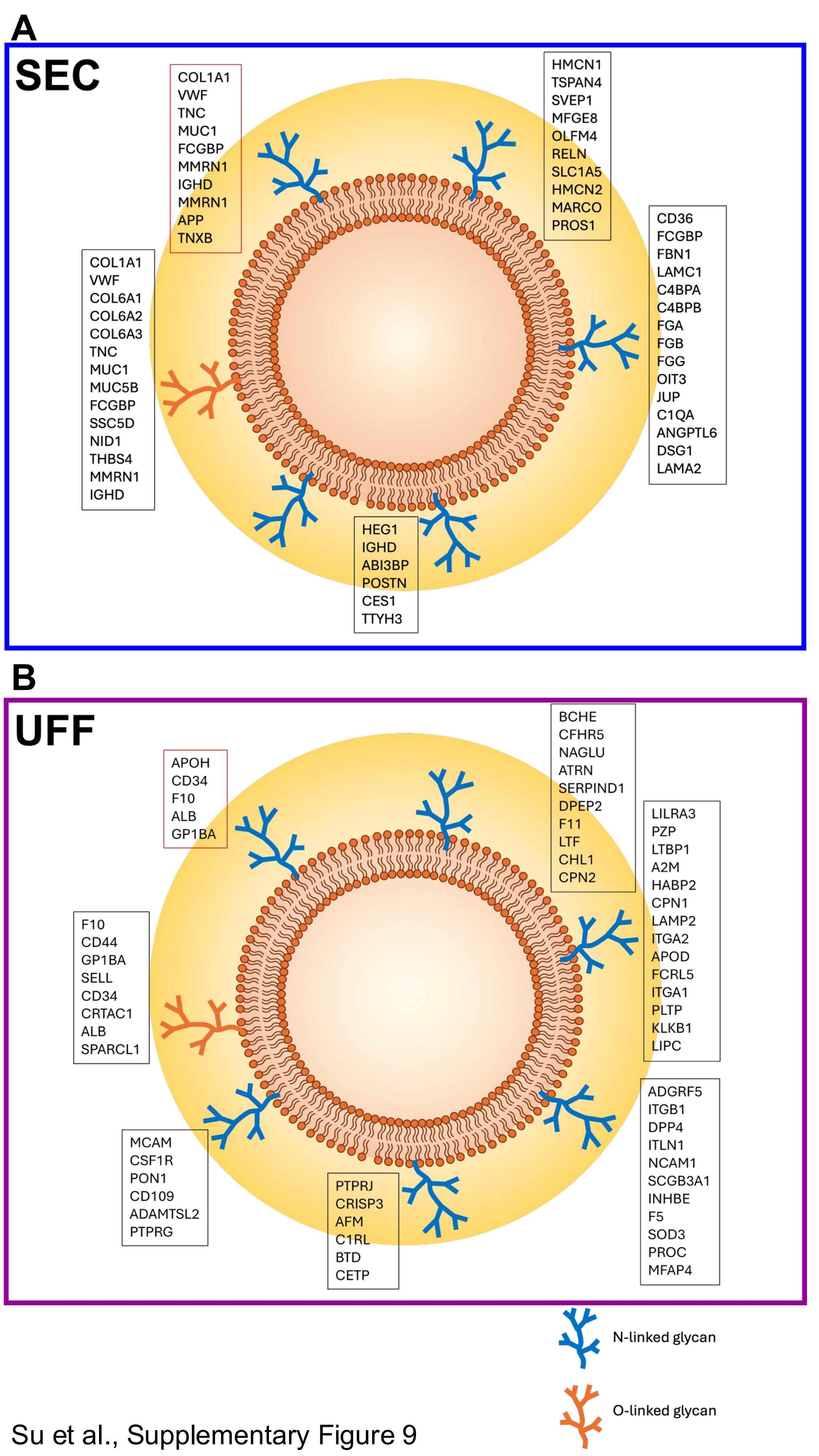

Supplement: Supplementary file 9 — Supplementary Figure 9. A graphical summary of glycoproteins significantly enriched in sEVs isolated by SEC and UFF, identified by mass spectrometry. (A) SEC and (B) UFF sEVs are denoted by the blue and purple outlined box, respectively. Glycoproteins of SEC and UFF sEVs are denoted by the black outlined box, glycoproteins that undergo both N‐glycosylation and O‐glycosylation are denoted by the red outlined box. [file JEV2-15-e70290-s008.jpg]

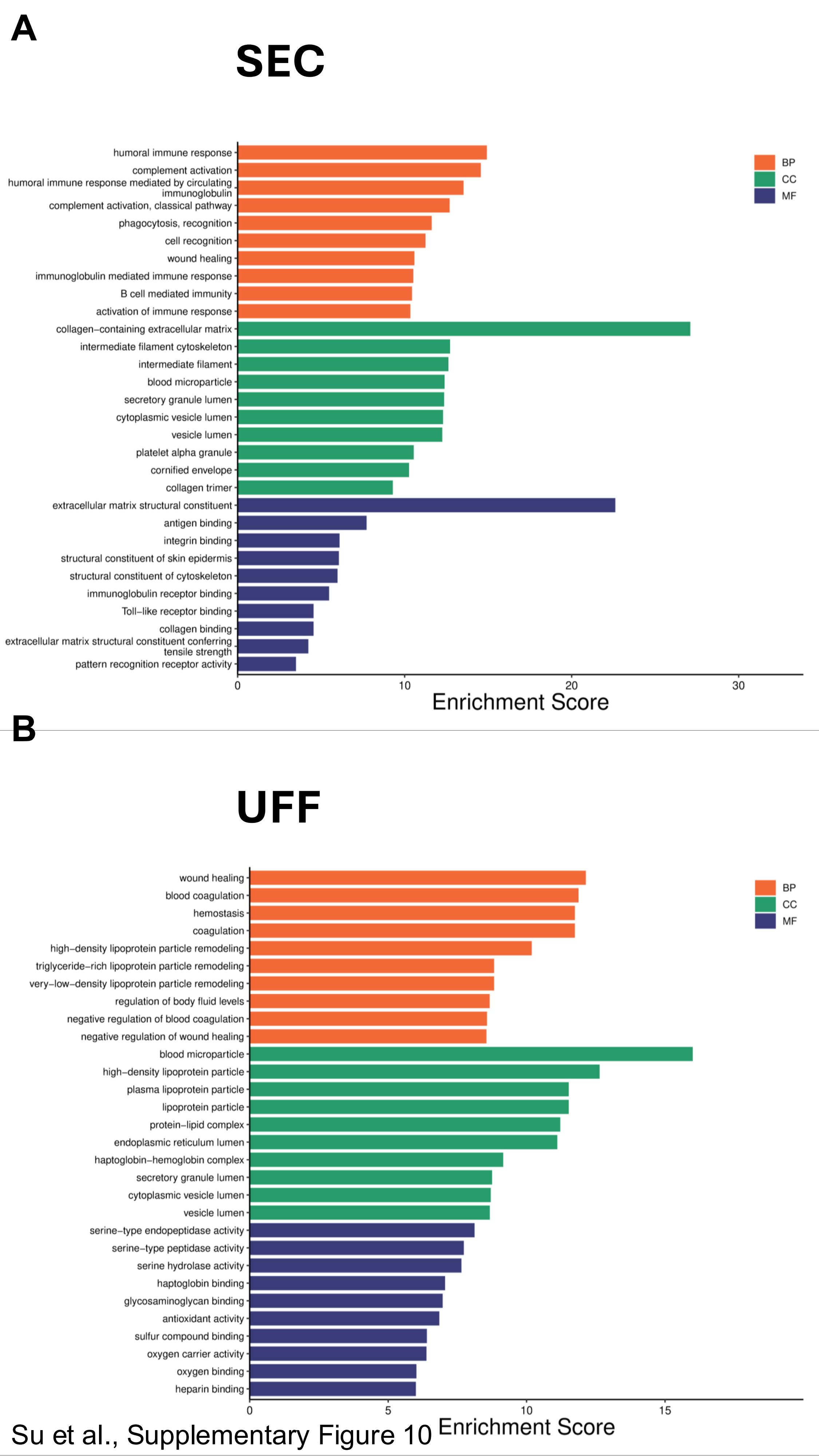

Supplement: Supplementary file 10 — Supplementary Figure 10. Gene Ontology analysis of proteins enriched in (A) SEC and (B) UFF sEVs. BP: biological process; CC: cellular component; MF: molecular function; SEC Size Exclusion Chromatography; UFF: Ultrafast Filtration. [file JEV2-15-e70290-s014.jpg]
